# Qualitative Detection Toward Military and Improvised Explosive Vapors by a Facile TiO_2_ Nanosheet-Based Chemiresistive Sensor Array

**DOI:** 10.3389/fchem.2020.00029

**Published:** 2020-01-31

**Authors:** Yushu Li, Wenyi Zhou, Baiyi Zu, Xincun Dou

**Affiliations:** ^1^Xinjiang Key Laboratory of Explosives Safety Science, Xinjiang Technical Institute of Physics & Chemistry, Urumqi, China; ^2^Key Laboratory of Functional Materials and Devices for Special Environments, Chinese Academy of Sciences, Urumqi, China; ^3^Center of Materials Science and Optoelectronics Engineering, University of Chinese Academy of Sciences, Beijing, China

**Keywords:** TiO_2_, chemiresistive sensor array, military explosives, improvised explosives, vapor detection

## Abstract

A facile TiO_2_ nanosheets-based chemiresistive gas sensor array was prepared to identify 11 kinds of military and improvised explosive vapors at room temperature. The morphology of TiO_2_ nanosheets was well-controlled by adjusting the concentration of HF applied during the preparation. Owing to the morphology difference, the TiO_2_ nanosheet-based sensors show different response values toward 11 kinds of explosives, which is the basis of the successful discriminative identification. This method owes lots of advantages over other detection techniques, such as the facile preparation procedure, high response value (115.6% for TNT and 830% for PNT) at room temperature, rapid identifying properties (within 30 s for 9 explosives), simple operation, high anti-interference property, and low probability of misinforming, and consequently has a huge potential application in the qualitative detection of explosives.

## Introduction

Rapid and accurate detection of explosives has been a hot issue of global concern due to the deepening terrorism crisis (Chen et al., 2010; Lichtenstein et al., 2014; Yang et al., 2015; Guo et al., 2017; Bastatas et al., 2018; Liu et al., 2019). The illegal blast induced by terrorists applied not only the powerful military explosives, but also the less powerful improvised explosives made of commercial available chemicals. Military explosives, mainly referred to nitro-explosives, such as 2,4,6-trinitrotoluene (TNT), dinitrotoluene (DNT), hexogen (RDX), and so on. The sensitive, selective, and rapid detection of nitro-explosive vapors is still a challenge owing to their low vapor pressures at room temperature. For instance, the room temperature saturated vapor pressures of TNT, DNT, para-nitro toluene (PNT), picric acid (PA), RDX are 9 ppb (part per billion), 180 ppb, 647 ppb, 0.97 ppb, and 4.9 ppt (part per trillion), respectively (Ewing et al., 2013). During the past decade, several techniques have been applied for the detection of military explosive vapors, such as fluorescence (Andrew and Swager, 2007; He et al., 2009; Olley et al., 2010; Zhu et al., 2011), surface enhanced Raman scattering (SERS) (Yang et al., 2010; Wang et al., 2014), ion mobility spectrometer (IMS) (Zhou et al., 2015), and chemiresistive sensors (Che et al., 2010; Chen et al., 2010; Engel et al., 2010; Wang et al., 2011a; Aluri et al., 2013). However, most of the previous reports were unable to realize the identification of different kinds of military explosives (Andrew and Swager, 2007; He et al., 2009; Che et al., 2010; Chen et al., 2010; Engel et al., 2010; Olley et al., 2010; Wang et al., 2011a; Zhu et al., 2011; Aluri et al., 2013; Zhou et al., 2015). Moreover, some of the reported techniques suffer from the extremely low response at room temperature (Chen et al., 2010; Aluri et al., 2013) and time consuming problem (Hutchinson et al., 2007; Zhu et al., 2011), inhabiting their application in the rapid on-the-spot detection of military explosives.

Different from the relative mature development of military explosive vapors detection, the detection of improvised explosives barely got any attention due to their ultra-low vapor pressure even at the typical maximum desorber temperature (Steinfeld and Wormhoudt, 1998; Mäkinen et al., 2011; Najarro et al., 2012; Peng et al., 2014). Improvised explosives are generally made of non-explosive compounds including KClO_3_, KNO_3_, KMnO_4_, S, NH_4_NO_3_, and urea (Kuila et al., 2006; Peters et al., 2015), *via* simple reaction or just blending, and are extensively used in terrorist attacks owing to their readily availability and low cost. Some techniques have been utilized for the detection of improvised explosives, such as capillary electrophoresis (CE) (Hutchinson et al., 2007; Blanco et al., 2011), ion chromatography (IC) (Dicinoski et al., 2006; Meng et al., 2008), and electrospray ionization mass spectrometry (ESIMS) (Zhao and Yinon, 2002; Flanigan et al., 2011). However, their drawbacks limited their application in the rapid identification of improvised explosives. For example, CE and IC need about 10 min to identify various kinds of anions and cations (Hutchinson et al., 2007; Johns et al., 2008), while ESIMS requires large equipment and therefore results in high testing expense and difficulties in on-the-spot application. Ionization mass spectrometry (IMS) has been proved to be an efficient technique for on-the-spot detection of trace improvised explosives such as KNO_3_, KClO_3_, and KClO_4_ within 5 s (Peng et al., 2014). However, it involves a time-consuming pretreatment procedure including sample swap and acidification. Therefore, a method to identify improvised explosives in a simple, fast, and low energy consuming manner is urgently needed.

Nanomaterial-based chemiresistive-gas sensor is an important explosives detection method due to the small device size, low energy consumption, high and rapid response (Senesac and Thundat, 2008; Che et al., 2010; Chen et al., 2010; Engel et al., 2010; Zu et al., 2013; Guo et al., 2014). For the detection of the military explosives, such as TNT, DNT, and RDX, several nanostructures have been explored as the sensing components, including TiO_2_(B) nanowires (Wang et al., 2011a), GaN/TiO_2_ heterostructure (Aluri et al., 2013), organic nanoribbons (Che et al., 2010), carbon nanotubes and ZnO nanowires (Chen et al., 2010). Moreover, nanostructured materials, such as Mn^2+^-doped ZnS nanocrystal, Fe-doped ZnO nanomaterial, and Aphen-doped TiO_2_ nanocrystal, have also been proved to be efficient for the gas sensing of improvised explosives (Qu et al., 2016; Wu et al., 2016; Xie and Liu, 2019). However, these chemiresistors can only detect a few explosive vapors, and the response values are as low as 5% at room-temperature (Chen et al., 2010; Aluri et al., 2013), leading to the increased possibility of misinforming.

In order to avoid the interference of other similar gases and decrease the misinformation, the chemically modified single-walled carbon nanotube (SWCNT) –based (Schnorr et al., 2013; Liu et al., 2015) and Si nanowire-based (Lichtenstein et al., 2014) nanosensor array were prepared to discriminatively identify different vapors. However, it is confirmed that the covalent functionalization of the SWCNTs can disrupt the extended electronic states and thus increase the base resistance, which may lower the sensitivity (Schnorr et al., 2013). Although quality sensors can be obtained by modest degree of functionalization, the experiment procedure is rather complicated (Bekyarova et al., 2010; Schnorr et al., 2013). In addition, the chemically modified selectors may increase the contact distance due to the existence of the functionalized molecular chains between analytes and the sensing materials, which may lower the sensor sensitivity. Nanostructured TiO_2_ is proved to be an efficient sensing material toward nitro-explosives detection (Wang et al., 2011a,b, 2013; Aluri et al., 2013; Tao et al., 2013; Yang et al., 2015). While doping with Apen, the TiO_2_ nanocrystal is applied for the detection of limited number of military and improvised explosives under UV-light illustration, including TNT, DNT, PA, S, AN, and TATP (Xie and Liu, 2019). However, the application of undoped TiO_2_ nanomaterials in the detection of improvised explosives remains unexplored. To the best of our knowledge, the morphology of nanomaterials has significant impact on their gas sensing performance since the geometric morphology difference can cause different specific surface area and the change in electron depletion layer (Gurlo, 2011; Cho et al., 2013). Hence, gas sensory array based on MoS2/RGO composites with various morphologies has been constructed for the recognitive detection of Triacetone Triperoxide (TATP) precursors (Sun et al., 2019). Furthermore, utilizing the fluorine as the capping agent for exposure facets stabilization, the morphology of TiO_2_ nanomaterials with treatment could be well tailored by modulating the synthesis parameters, including F sources, the concentration of the source, reaction temperature and time, and so on (Lee et al., 2016; Yan et al., 2017; Zhao et al., 2017). However, there is no attempt on the construction of gas sensor array based on nanostructured TiO_2_ with different morphologies to realize the discrimination of various explosives.

In this work, a series of TiO_2_ nanosheets with different morphologies were successfully prepared via the hydrothermal reaction with the help of F^−^. The gas sensor array with these TiO_2_ nanosheets as the sensing components can identify the 5 nitro-explosive vapors (TNT, DNT, PNT, RDX, PA) and 6 improvised explosive vapors (including KNO_3_, KClO_3_, KMnO_4_, S, NH_4_NO_3_, urea) successfully.

## Materials and Methods

### Chemicals

Tetrabutyl orthotitanate (TBOT), concentrated sulfuric acid (H_2_SO_4_, 98%), hydrofluoric acid (HF, 40%), 2,4-dinitrotoluene (DNT), *p*-nitrotoluene (PNT), picric acid (PA), potassium nitrate (KNO_3_), potassium chloride (KClO_3_), potassium permagnate (KMnO_4_), sulfur (S), ammonium nitrate (NH_4_NO_3_), and urea were purchased from Sigma-Aldrich. 2,4,6-trinitrotoluene (TNT) and hexogen (RDX) were obtained from the National Security Department of China. Except for TNT was recrystallized with ethanol before use, all other chemicals were of analytical grade and used without further purification.

#### Caution

TNT and other nitro-explosives used in the present study are highly explosive and should be handled only in small quantities.

### Preparation of TiO_2_ Nanosheets

The TiO_2_ nanosheets were prepared via a hydrothermal method. In a typical procedure, different amounts of HF (0–1 ml) were added to the mixture of 12.5 ml TBOT and 1.5 ml H_2_SO_4_ with vigorous stirring, followed by the addition of certain amount of H_2_O to maintain the total volume of the reaction mixture as 15 ml. The mixture was then transferred to Teflon lined autoclave and kept at 180°C for 24 h. After completion of the reaction, the white precipitate was filtered and washed with ethanol several times and then dried in air at 60°C.

### Characterization

X-ray diffraction (XRD) measurement was conducted using powder XRD (Bruker D8 Advance, with Cu-K_α_ radiation operating at 40 kV and 40 mA, scanning from 2θ = 10 to 90°). Field-emission scanning electron microscopy (FESEM, ZEISS SUPRA 55VP), and transmission electron microscope (JEM-2011 TEM, 200 kV) were used to characterize the morphology and the detailed structure of the samples.

### Sensor Array Fabrication and Gas Sensing Performance Testing

The obtained TiO_2_ nanosheets were mixed with deionized water in a weight ratio of 4:1 and ground in a mortar for 10 min to form a uniform paste. The paste was then coated on a ceramic substrate, on which silver interdigitated electrodes with both finger-width and inter-finger spacing of about 200 μm was previously printed, by a thin brush to construct a gas sensor. The thickness of the film was controlled by the brushed cycles. The sample was dried naturally in air overnight and aged at 10 V in air to ensure the good stability. Five gas sensors from TiO_2_ with different morphologies were fabricated together to construct the sensor array. The room temperature-saturated explosive vapor was obtained by putting solid explosive powder (1 g) at the bottom of a conical flask (50 mL) before it was sealed for 48 h. All tests were performed at consistent operating temperature (room temperature, 25 ± 2°C) and relative humidity (30 ± 3%) to avoid undesired signal fluctuate. For gas sensing test, the sensor was inserted into the saturated vapor of an explosive. After the sensor resistance reached a new constant value, the sensor was then inserted into a same size conical flask full of air to recover. The electric signal (current) of the sensor was recorded by electrochemical workstation (CIMPS-2, ZAHNER). The essential gas sensing characteristics, namely the corresponding response value, response time and recovery time, can be obtained from the response curves. The response value is the steady-state value of the response with exposure toward explosive vapors, and is defined as,

(1) $$\begin{array}{r} \text{Response}=\frac{I_{g}-I_{a}}{I_{a}}*100\% \end{array}$$

where I_g_ and I_a_ are the current value of the gas sensor measured in explosive vapor and in air at room temperature, respectively. The response time is defined as the period it takes to cause 90% of the current changes upon exposure to the explosive vapor, while the recovery time is defined as the period it takes to cause 90% of the current changes after the explosive vapor is removed.

## Results and Discussion

### Morphology Tailoring of TiO_2_ Nanosheets

**Figure 1:**
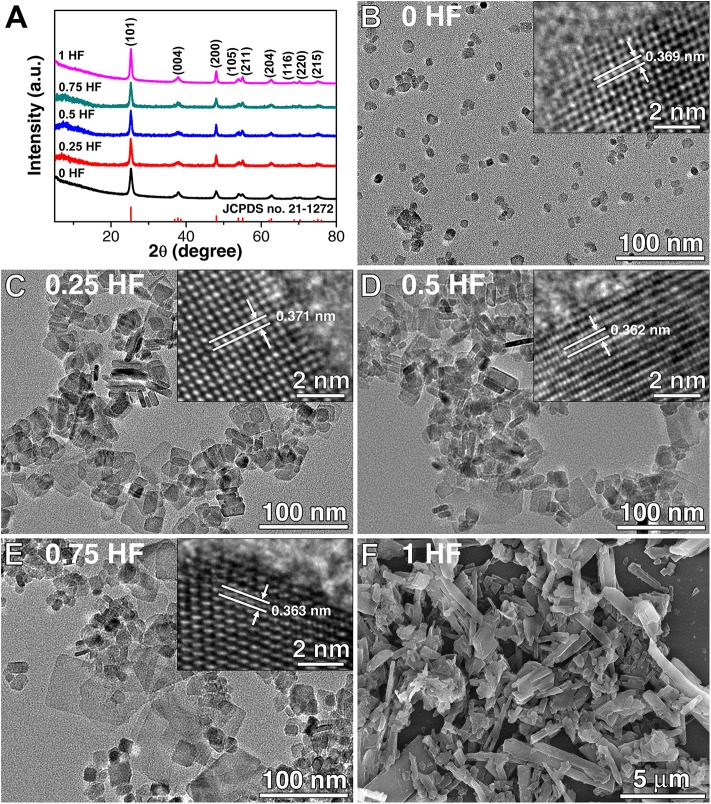
**(A)** XRD patterns of five kinds of TiO_2_ nanosheets, TEM, and SEM images of the TiO_2_ nanosheets prepared with different amounts of HF **(B)** 0 ml, **(C)** 0.25 ml, **(D)** 0.5 ml, **(E)** 0.75 ml, and **(F)** 1 ml (insets are HRTEM images).

X-ray diffraction (XRD) analysis was performed to investigate the crystal phase of the TiO_2_ nanosheets prepared by adjusting the amounts of HF solution (0–1.0 ml) applied in the hydrothermal reaction. As shown in Figure 1A, it is obvious that all the diffraction peaks of TiO_2_ nanosheets prepared via this method can be well-indexed as the anatase TiO_2_ phase (JCPDS NO. 21-1272), demonstrating that the phase of the TiO_2_ nanosheets would not be affected by the concentrations of HF solution within this range during the preparation. The morphology of the TiO_2_ nanosheets was investigated by transmission electron microscopy (TEM) and field emission scanning electron microscopy (FESEM), as shown in Figures 1B–F. It is found that the morphologies are different with the increasing amount of HF applied from 0 to 1.0 ml. With the absence of HF, the TiO_2_ nanoparticles grew randomly and form the irregular shape with size ranging from 5 to 20 nm, and there is no continuous growth of large nanosheets (Figure 1B). With the amount of HF increasing to 0.25 ml, it is observed that the TiO_2_ crystals grew larger to form rectangular-shaped nanosheets, which consist of the smaller ones with size of 15–25 nm as major and limited number of larger nanosheets over 40 nm (Figure 1C). With the amount of HF continuously increasing to 0.50 ml, the number of relatively larger sized TiO_2_ nanosheets (45 nm in average) increased, however, the smaller nanosheets (20 nm in average) are still the major component due to the restricted HF amount (Figure 1D). Meanwhile, it is also observed that extra-large nanosheets with size about 100 nm start to form. With the amount of HF continuously increasing to 0.75 ml, although there are still smaller nanosheets (20 nm) and larger sized TiO_2_ nanosheets (45 nm in average) existing, the extra-large nanosheets with size around 100 nm are distinctively observed (Figure 1E). When the amount of HF further increases to 1.0 ml, the overgrowth of TiO_2_ nanosheets occurred leading to the sheet-like structures with size of several microns (Figure 1F). Hence, with the amount of HF increasing from 0 to 1 ml, the size of TiO_2_ nanosheets grew from a dozen nanometers to several microns. High resolution transmission electron microscopy (HRTEM) was adopted to obtain the detailed crystal structure (insets in Figures 1B–F), clear lattice fringes with the lattice spacing corresponding to the (101) plane of anatase TiO_2_ were shown, indicating the good crystallinity of the samples. As a whole, the increased HF content in the original reaction mixture promotes the growth of the anatase TiO_2_ nanosheets with more reactive facets beneficial for sensing due to the enlarged capping effect. This phenomenon is in good agreement with the previously reported results, in which the growth behavior of TiO_2_ with various contents of F^−^ is systematically studies (Lee et al., 2016). Therefore, the introduction of F^−^ ion during the preparation is a reasonable choice to control the growth, tailor the morphology, and thus adjust the sensing performance of anatase TiO_2_ nanosheets.

### Varying Sensing Performance Toward Military and Improvised Explosive Vapors

**Figure 2:**
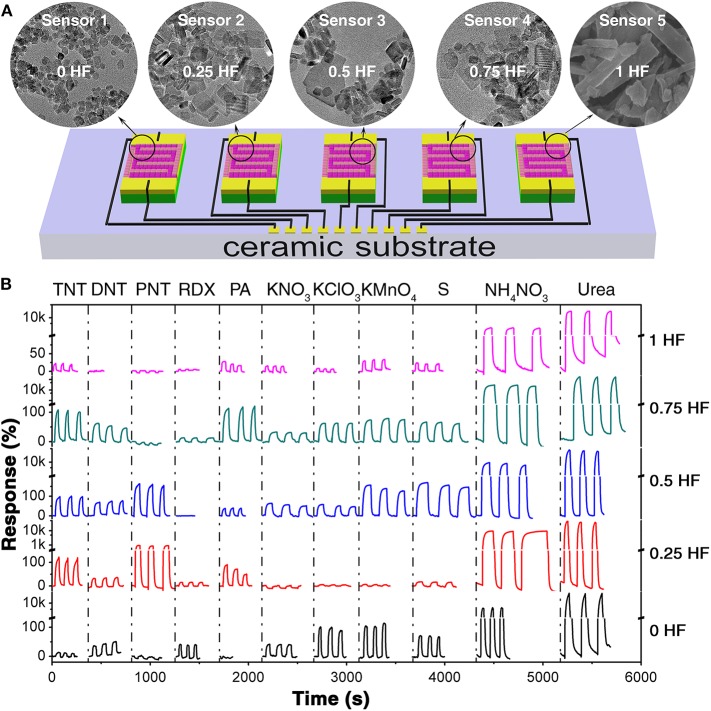
**(A)** The schematic diagram of the gas sensor array based on TiO_2_ nanosheets. **(B)** The corresponding response curves toward 11 explosive vapors (TNT, DNT, PNT, RDX, PA, KNO_3_, KClO_3_, KMnO_4_, S, NH_4_NO_3_, and urea).

The as-prepared TiO_2_ nanosheets with different morphologies were fabricated on ceramic substrate with comb-like electrodes, respectively, to construct a chemiresistive gas sensor array, as schematically shown in Figure 2A. The obtained sensor array was exposed to five saturated military explosive vapors (TNT, DNT, PNT, RDX, PA) and six saturated improvised explosive vapors (KNO_3_, KClO_3_, KMnO_4_, S, NH_4_NO_3_, urea) at room-temperature for evaluation of the sensing properties (Figure 2B). The room-temperature saturated vapor pressures of all these analytes are extremely low, such as 9 ppb for TNT, 411 ppb for DNT, 647 ppb for PNT, 0.97 ppb for PA, 4.9 ppt for RDX, 2 ppb for S, 9 ppt for urea, and 14.7 ppb for NH_4_NO_3_ (Lyons, 2011; Ewing et al., 2013). Meanwhile, the other three explosives, KNO_3_, KClO_3_, and KMnO_4_, owing to their ionic crystal nature, are non-volatile and hard to decompose at room temperature indicating that neither the vapor of themselves nor their decomposition products is responsible for the gas sensing signal (Supplementary Table 1) However, it has been discovered that microparticulates could be separated from non-volatile solids and suspended in air (Clark and Shirley, 1973; Samet et al., 2004; Li et al., 2018; Yao et al., 2018). Therefore, we believe that the microparticulates suspended in the vapor of these explosives, which could interact with the surface of sensing materials and hence are responsible for the electric signal changes of the sensors in the array. The response curves are generated from the current change traces of the sensors toward explosive vapors at an applied voltage of 10 V. From the current change behaviors (Supplementary Figures 1–10), it is obvious that with the immersing of the sensor array into explosive vapors, the resistances change immediately, and then with the immersing of the sensor array into air, the resistance change back to its initial value rapidly, indicating the good repeatability of the sensor array toward each explosive vapor. It is also observed that each sensor in the array shows different resistance change with exposure to different kinds of explosive vapors. While with immersion into the same explosive vapor, the sensors in the array show different resistance change as well. It is believed that the resistance change of the TiO_2_ nanosheets sensing materials was caused by the change of the charge depletion layer depth. For a single sensor in the array exposed to various explosive vapors, the different gas molecules adsorbed on the surface of TiO_2_ nanosheets would lead to different surface potential barrier, which depends on the charge density established upon interaction between the adsorbed target gas and active sites on the surface of the sensing layer. Thus, different charge depletion layer depth would be resulted and hence the difference in resistance was observed. Furthermore, the differences in response among all sensors in the array toward the same explosive vapor are caused by the different charge depletion layer depth introduced by morphology tailoring. The modulation of sensing performance by morphology tailoring could be attribute to the capping effect of F^−^ ion. It is observed that the response of the TiO_2_ nanosheets toward certain explosive vapor generally increased first and then decrease with the increasing amount of F^−^ ion. On one hand, since the F^−^ ion serves as the capping agent for stabilizing reactive facets (Lee et al., 2016), with the increasing amount of F^−^ ion, more reactive facets are exposed for sensing, resulting in the enhanced sensing performance. While on the other hand, with the increasing amount of F^−^ ion, the TiO_2_ nanosheet crystals grow larger, leading to a reduced charge depletion layer depth which is hindering the sensing performance enhancement. However, due to the extreme complicated gas sensing response process, the responses could be affected by many factors, including the interaction between the analyte and the sensing material, decomposition products, the humidity change caused by the analyte and the floating tiny clusters of the analyte (Supplementary Table 1), and hence they are not strictly in line with the changing trend. Therefore, the sensing performance of TiO_2_ nanosheets toward explosive vapors could be modulated by morphology tailoring.

**Figure 3:**
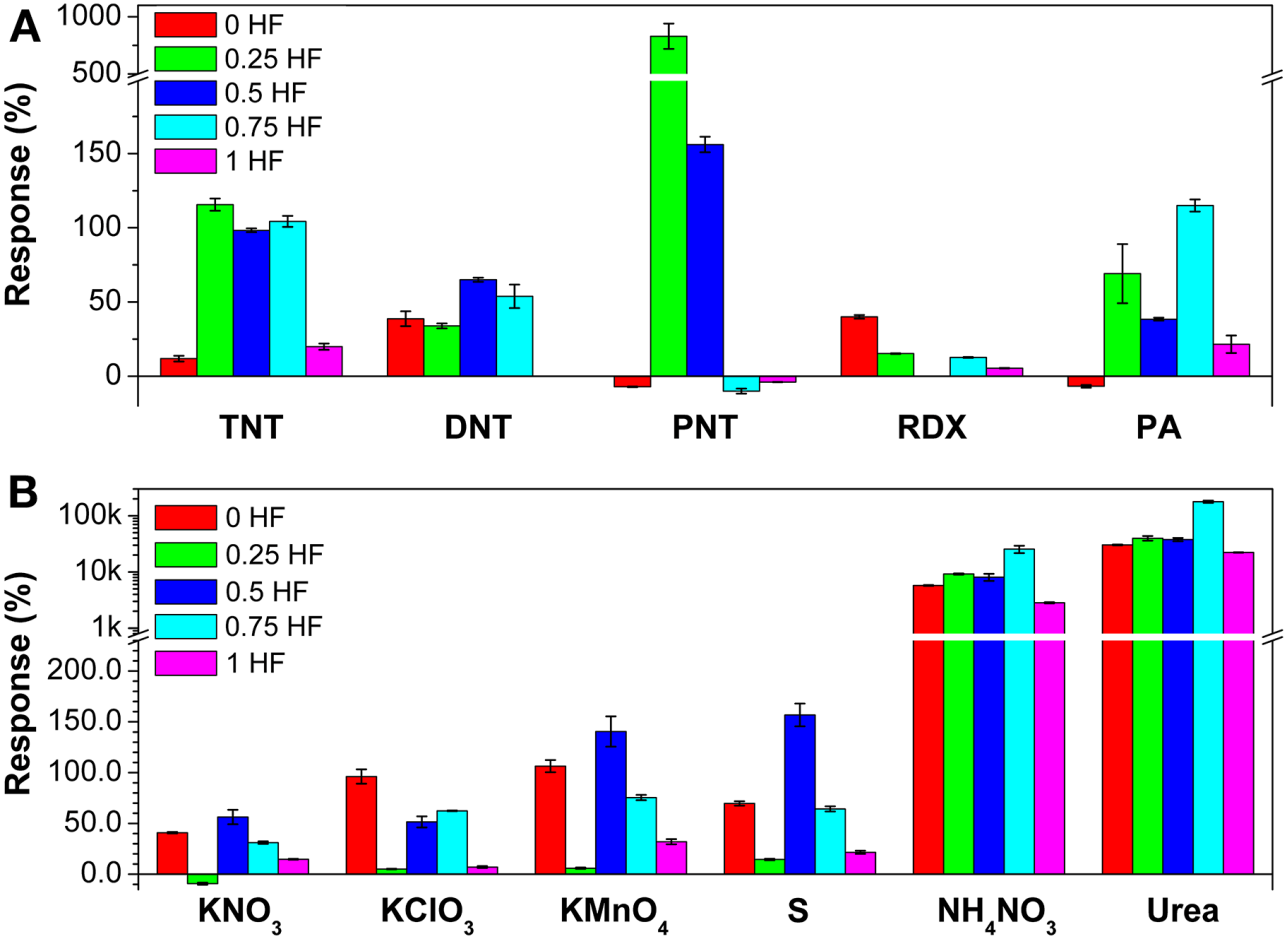
The responses of the gas sensor array toward **(A)** 5 military explosive vapors and **(B)** 6 improvised explosive vapors.

The response values of the sensor array toward 5 kinds of military explosive vapors and 6 kinds of improvised explosive vapors are summarized from the response curves (Figure 3). It is clearly shown that all 5 gas sensors consisted in the array can detect 11 kinds of explosive vapors yielding different response values. For military explosives detection (Figure 3A), such as, toward TNT and PNT vapors, sensor 2 (0.25 HF) shows the largest responses of 115.6 and 830.0%, respectively. Sensor 3 (0.5 HF) shows the largest response value (65.0%) toward DNT. Toward RDX, sensor 1 (0 HF) shows the largest response of 40.0%. Sensor 4 (0.75 HF) shows the largest response value of 115.0% toward PA. Toward improvised explosives, the TiO_2_ nanosheet-based gas sensor array also show excellent gas sensing performance (Figure 3B). Sensor 3 (0.5 HF) exhibits the largest responses toward KNO_3_, KMnO_4_ and S, while the corresponding values are 56.3, 140.5, and 156.8%, respectively. Sensor 1 (0 HF) exhibits the largest response of 96.2% toward KClO_3_. Toward NH_4_NO_3_ and urea, all sensors show strikingly large responses, and sensor 4 (0.75 HF) exhibits the largest responses of 255.6 and 1783.1 times, respectively. To sum up, TiO_2_ nanosheets with different morphologies show different response values toward nitro- and improvised explosive vapors, which further indicates that the sensing performance of TiO_2_ nanosheets can be well-regulated to achieve the response differences by simply controlling the morphology. Furthermore, it should be noted that the TiO_2_ nanosheet-based sensor array can work in a very large response range, from zero to a few thousands, which would enhance the practical application of the array.

**Figure 4:**
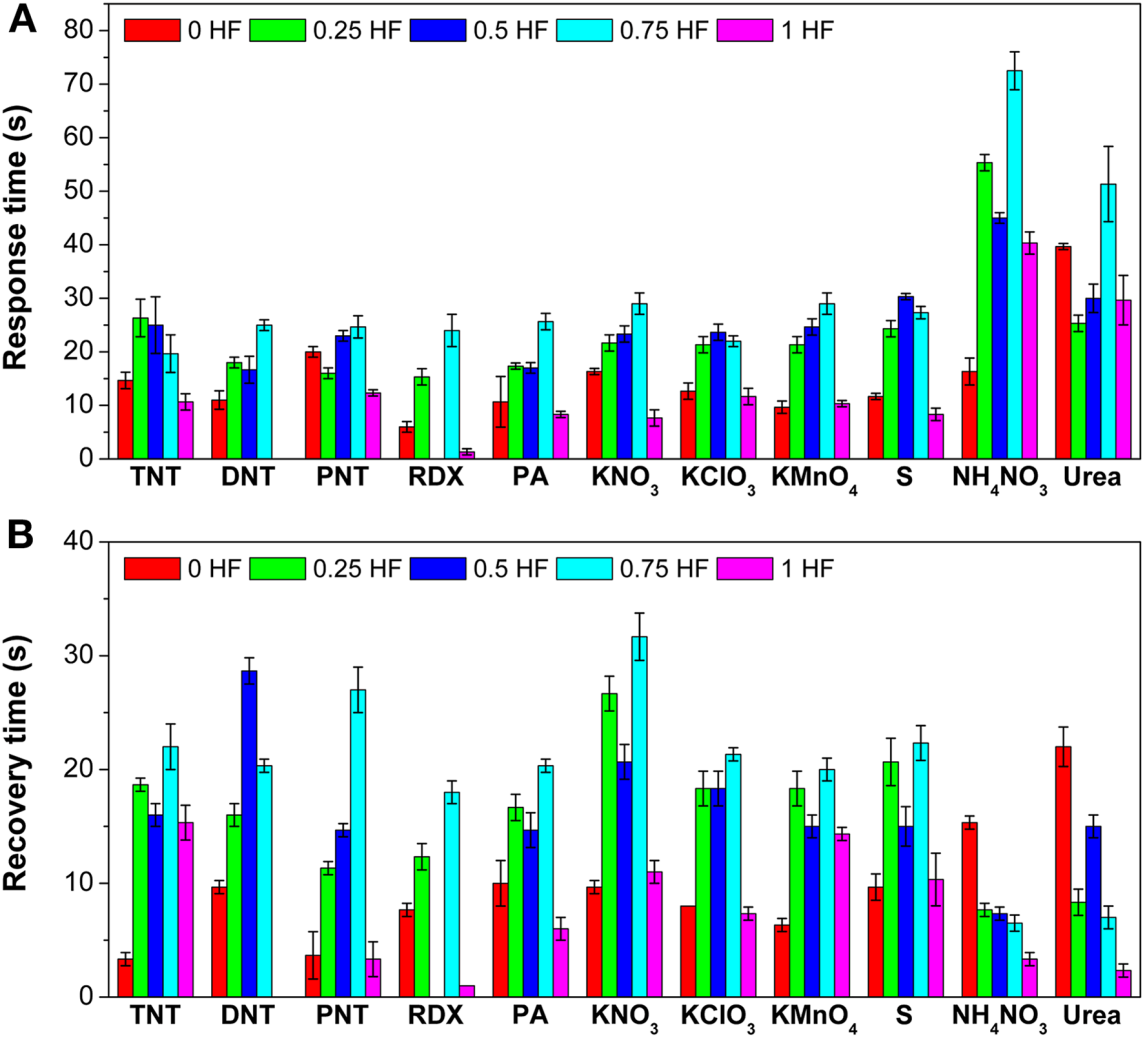
**(A)** Response times and **(B)** recovery times of the gas sensor array toward 11 explosive vapors.

Besides the response values, response time and recovery time of the gas sensor array toward 11 explosive vapors are also summarized (Figure 4). Toward TNT, PNT, RDX, PA, KNO_3_, KClO_3_, and S, sensor 5 (1 HF) shows the fastest response with response times of 10.7, 12.3, 1.3, 8.3, 7.7, 11.7, and 8.3s, respectively (Figure 4A). Meanwhile, toward DNT, KMnO_4_, and NH_4_NO_3_, sensor 1 (0 HF) shows the fastest response, and the corresponding response times are 11.0, 9.7, and 16.3 s, respectively. It should be noted that the response time of sensor 2 (0.25 HF) toward urea is only 25.3 s although the response value is as high as 399.5. The response time periods are within 30 s for most of the explosive vapors.

Similar as the response time, the recovery time of sensors in the array are also different resulting from the morphology differences of TiO_2_ nanosheets (Figure 4B). Toward TNT, DNT, PNT, KNO_3_, KClO_3_, KMnO_4_, and S, sensor 1 (0 HF) shows the fastest recovery, and the corresponding recovery times are 3.3, 9.7, 3.7, 9.7, 8.0, 6.3, and 9.7 s, respectively. Toward RDX, PA, NH_4_NO_3_, and urea, sensor 5 (1 HF) shows the fastest recovery with recovery times of 1.0, 6.0, 3.3, and 2.3 s, respectively. As a whole, the TiO_2_ nanosheet-based gas sensor array can recover to its initial state within 35 s in atmosphere, indicating that it can be employed for the next detection cycle rapidly.

**Figure 5:**
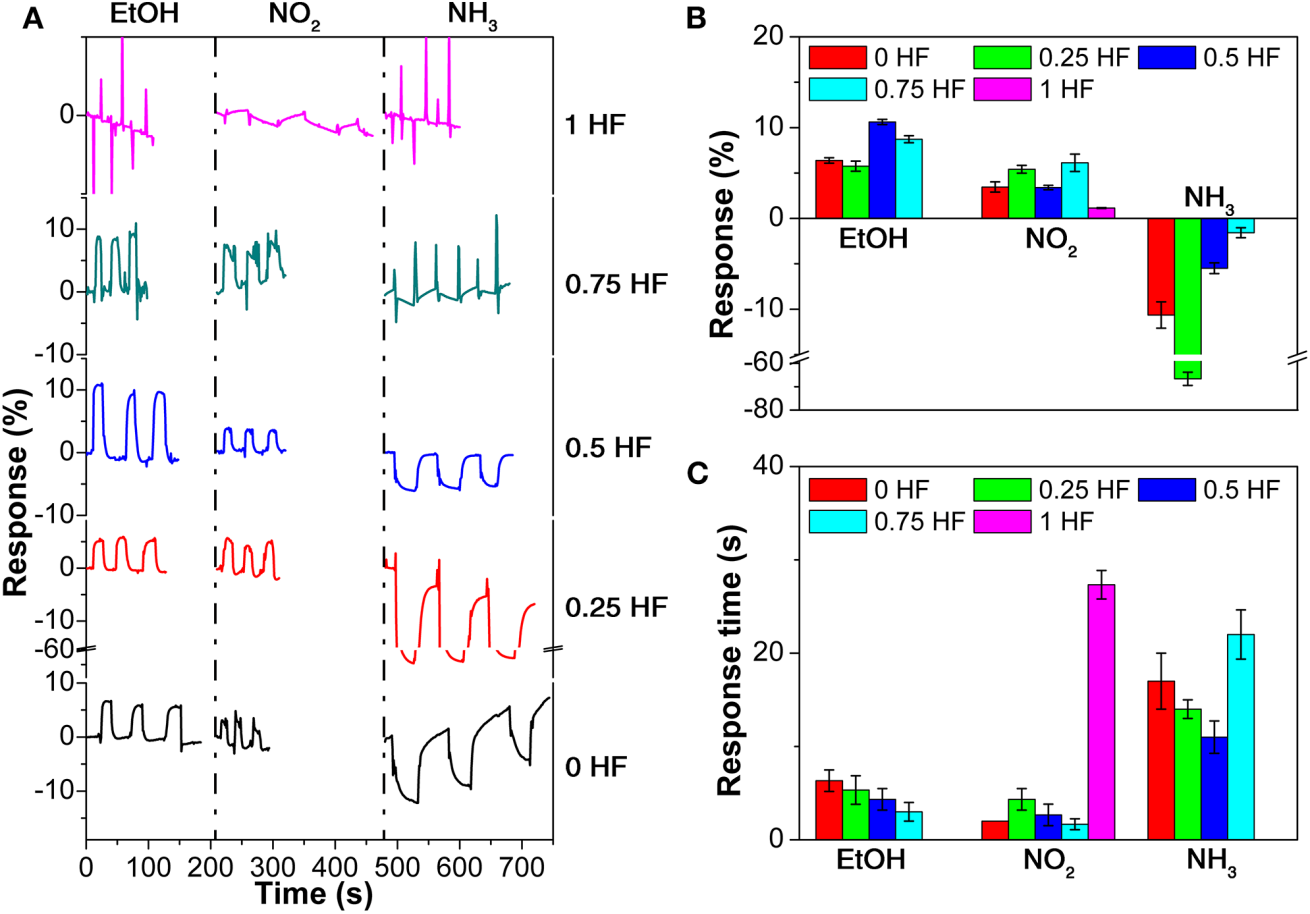
**(A)** Response curves, **(B)** response values, and **(C)** response time of the gas sensor array toward 3 interfering gases.

The anti-interfering performance of the sensor array is evaluated by exposing the sensor array to common interfering gases, namely ethanol (EtOH), NO_2_, and NH_3_, with concentration of 1 ppm (Figure 5; Supplementary Figures 11–15). All 5 gas sensors in the array exhibit positive response toward EtOH and NO_2_ gases and negative response toward NH_3_ (Figure 5A), which is in good agreement with results discussed above. It is worth to notice that the currents of the TiO_2_ nanosheets based sensors increase in typical electron withdrawing target vapors, such as TNT, DNT, and NO_2_, which is rare and under further exploration. The response values toward EtOH and NO_2_ are much smaller compared with that of explosive vapors. For instance, sensor 3 (0.5 HF) shows the largest response of 10.6% toward EtOH, while sensor 4 (0.75 HF) shows the largest response of 6.1% toward NO_2_ (Figure 5B). Meanwhile, in the case of NH_3_, the sensors in the array all shows negative response values with a largest response of −66.7%, which is relatively large but in the opposite direction compared with that of explosive vapors. The response time toward these interfering gases are all <30 s which are comparable with that of the explosive vapors (Figure 5C). Considering the fact that the room temperature vapor pressures of the explosives are much lower than 1 ppm, it can be concluded that the common interfering gases in real-world have limited influence on the sensing performance of the sensor array toward explosive vapors.

### Discriminative Recognition of Explosive Vapors

**Figure 6:**
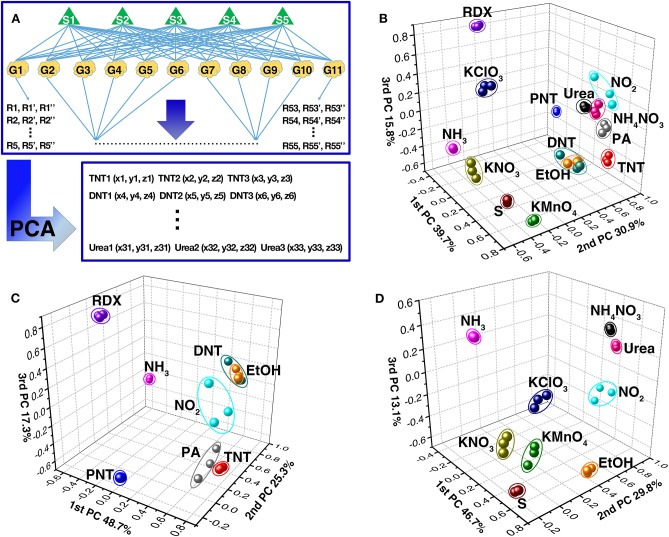
**(A)** The schematic diagram of the data analysis using PCA. PCA plots of **(B)** 11 kinds of different explosives, **(C)** military explosives, and **(D)** improvised explosives along with three interfering gases from the responses of the TiO_2_ nanosheet-based gas sensor array.

The methodology of principal component analysis (PCA), which could extract the selective feature of original data depending on variance criteria and visualize the extracted feature, was used to discriminatively recognize explosives. Its analysis procedure is schematically shown in Figure 6A. All responses data from the gas sensor array were subjected to PCA, and were transformed to a new coordinate system. Afterwards, each kind of explosive vapor can be represented as a point in the new three-dimensional space, such as TNT1 (x1, y1, z1). Thus, different explosives and the interfering gases appear at different positions in the new coordinate system, as shown in Figures 6B–D.

Based on the response difference induced by morphology difference between TiO_2_ nanosheets, all 11 kinds of explosives and 3 interfering gases can be discriminatively identified except for DNT and EtOH, which are overlapping, and urea and NH_4_NO_3_, which are very close to each other (Figure 6B). If only the family of military explosives and the interfering gases are considered, the clusters of organic explosives and some of the interfering gases are scattered further from each other with the exception of DNT and EtOH (Figure 6C). Similarly, if only the family of improvised explosives and the interfering gases are of interest, the clusters of improvised explosives are also scattered further from each other, such as KNO_3_, KClO_3_ KMnO_4_, S, NH_3_, NO_2_, and EtOH from NH_4_NO_3_ and urea (Figure 6D). However, it should be noted that although the PCA analysis of responses is powerful enough to discriminate 10 explosives and 2 interfering gases, it is difficult to discriminate DNT from EtOH and urea from NH_4_NO_3_ using PCA analytical method based only on the response values.

**Figure 7:**
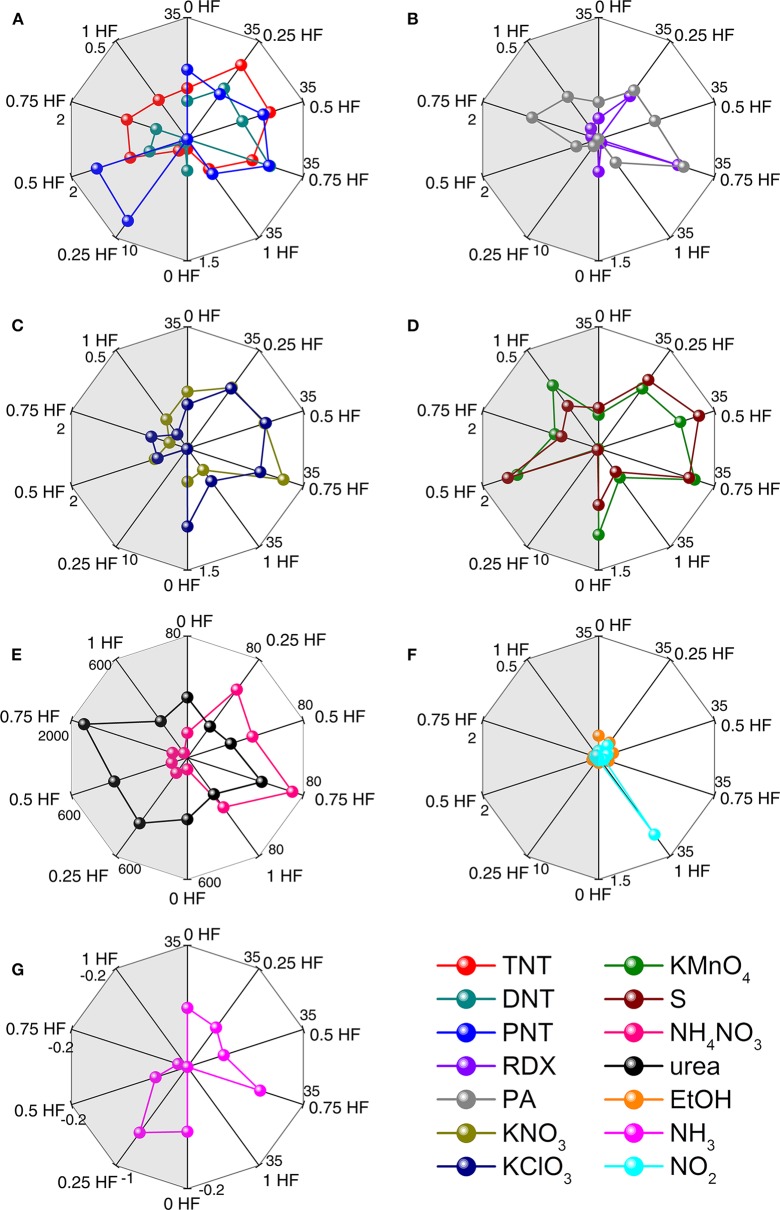
Radar plots of response values and response times of 11 explosives and 3 interfering gases: **(A)** TNT, DNT, and PNT; **(B)** RDX and PA; **(C)** KNO_3_ and KClO_3_; **(D)** KMnO_4_ and S; **(E)** NH_4_NO_3_ and urea; **(F)** EtOH and NH_3_; **(G)** NO_2_. Left side response value represents the thermodynamically derived results for each analyte, and the right side response time represents the kinetically derived results for each analyte.

During the gas sensing procedure, the response value as a steady state parameter is associated with the thermodynamic interaction between the explosive species and the TiO_2_ nanosheets, while the response time and recovery time are associated with the kinetic interaction between them. However, response time is a more meaningful kinetic parameter for explosives recognition in practical detecting situation since fast responding is essential to achieve early alarming. Therefore, by combining the rapid mathematical analysis of the thermodynamic and kinetic interactions, namely the response value and response time, a visible fingerprinting method is utilized to realize the discriminative identification of explosives. This fingerprinting method is suitable for discriminative identification as the shape of the fingerprinting pattern is independent with the analyte concentration (Lichtenstein et al., 2014). Figure 7 shows the fingerprinting radar plot patterns of all explosive vapors and 3 interfering gases generated from the response values and response times of the sensors in the array. It is obvious that each analyte has its unique fingerprinting pattern, which can be used to distinguish from each other and thus to realize the discriminative identification of all the analytes including not only the explosives but also the interfering gases (Figures 7A–G). For instance, TNT, DNT, and PNT exhibit different patterns although they have similar molecular structures. Meanwhile, although KNO_3_ and KClO_3_ are both potassium salts, their radar plot patterns distinguish from each other. This distinction should be ascribed to the different interactions between different explosive vapors and TiO_2_ nanosheets with different morphologies. In the case of NH_4_NO_3_ and urea, which are hard to be discriminated using PCA method, they can be differentiated from each other easily owing to their remarkably different radar plot patterns. While for DNT and EtOH, which are unable to be distinguish in the PCA plot, the radar plot patterns are of great difference and thus it is also easy to discriminate them from each other using this method. Therefore, with straightforward data analysis, the chemiresistive sensor array from TiO_2_ nanosheets with different morphologies is capable of discriminatively identifying 11 types of explosive vapors and 3 common interfering gases.

Furthermore, the present chemiresistive gas sensor array has lots of advantages for practical application. Compared with other vapor electrical sensors (Che et al., 2010; Chen et al., 2010; Wang et al., 2011a; Aluri et al., 2013; Schnorr et al., 2013; Liu et al., 2015; Yang et al., 2015), it shows a higher sensitivity toward TNT, DNT, and PNT, and can detect RDX vapor at ppt level (Supplementary Table 2). Compared with other military explosive detecting techniques (Andrew and Swager, 2007; He et al., 2009; Olley et al., 2010; Zhu et al., 2011; Wang et al., 2014; Zhou et al., 2015), it can identify 5 military explosives within 30 s and avoid (1) the interference induced by other substances, (2) the large and expensive instrumentation, and (3) complicated operating procedure, which might be problematic for fluorescence, SERS, and IMS (Supplementary Table 3). In the case of detection of improvised explosives, the present gas sensor array can discriminatively identify 6 improvised explosive vapors within 75 s, which is much more efficient compared with CE and IC (Supplementary Table 4; Hutchinson et al., 2007; Peng et al., 2014). Moreover, it can avoid complicated operation which is essential in IMS technique (Johns et al., 2008). Therefore, the present TiO_2_ nanosheet-based chemiresistive gas sensor array shows high sensitivity, short testing time, handy operation, and the ability to avoid interference, which are beneficial for practical application in rapid identification of military and improvised explosives.

## Conclusions

A series of TiO_2_ nanosheets with well-tailored morphologies were successfully prepared via a simple hydrothermal method. HF was utilized as morphology modulation agent in the reaction. The size of the TiO_2_ nanosheets grew larger with the increase of the amount of HF. The morphological difference of TiO_2_ nanosheets leads to the dissimilarity of the specific surface area and the charge depletion layer depth, and hence different responses toward explosive vapors. The gas sensor array based on the series of TiO_2_ nanosheets can rapidly and discriminatively identify the vapors of 5 nitro-explosives (TNT, DNT, PNT, RDX, PA) and 6 improvised explosives (KNO_3_, KClO_3_, KMnO_4_, S, NH_4_NO_3_, urea) along with 3 common interfering gases (EtOH, NO_2_, NH_3_) successfully under room-temperature condition with the help of PCA and fingerprinting pattern recognition method. It has a huge potential for practical application owing to its obviously superior advantages compared with other detection techniques. Thus, this work presents an efficient method to achieve the response differences simply by the morphology tailoring, and consequently to realize the identification of nitro- and improvised explosives, which is an important attempt for the development of quality sensor array for explosive detection.

## Data Availability Statement

The raw data supporting the conclusions of this article will be made available by the authors, without undue reservation, to any qualified researcher.

## Author Contributions

All authors listed have made a substantial, direct and intellectual contribution to the work, and approved it for publication.

### Conflict of Interest

The authors declare that the research was conducted in the absence of any commercial or financial relationships that could be construed as a potential conflict of interest.
