# Supplementary material for: Qualitative Detection Toward Military and Improvised Explosive Vapors by a Facile TiO2 Nanosheet-Based Chemiresistive Sensor Array
Source: Front Chem. 2020 Jan 31;8:29. doi: 10.3389/fchem.2020.00029 (PMC7005537; doi:10.3389/fchem.2020.00029)
Supplement: Supplementary file 1 [file Table_1.docx]

Supplementary Material

**Qualitative Detection towards Military and Improvised Explosive Vapors by a Facile TiO_2_ Nanosheet-based Chemiresistive Sensor Array**

Yushu Li ^1#^, Wenyi Zhou ^1,2#^, Baiyi Zu ^1^, Xincun Dou ^1,2*^

^1^ Xinjiang Key Laboratory of Explosives Safety Science, Xinjiang Technical Institute of Physics & Chemistry; Key Laboratory of Functional Materials and Devices for Special Environments, Chinese Academy of Sciences, Urumqi 830011, China.

^2^ Center of Materials Science and Optoelectronics Engineering, University of Chinese Academy of Sciences, Beijing 100049, China.

[xcdou@ms.xjb.ac.cn](mailto:xcdou@ms.xjb.ac.cn)

Includes:

15 supplementary figures

3 supplementary tables


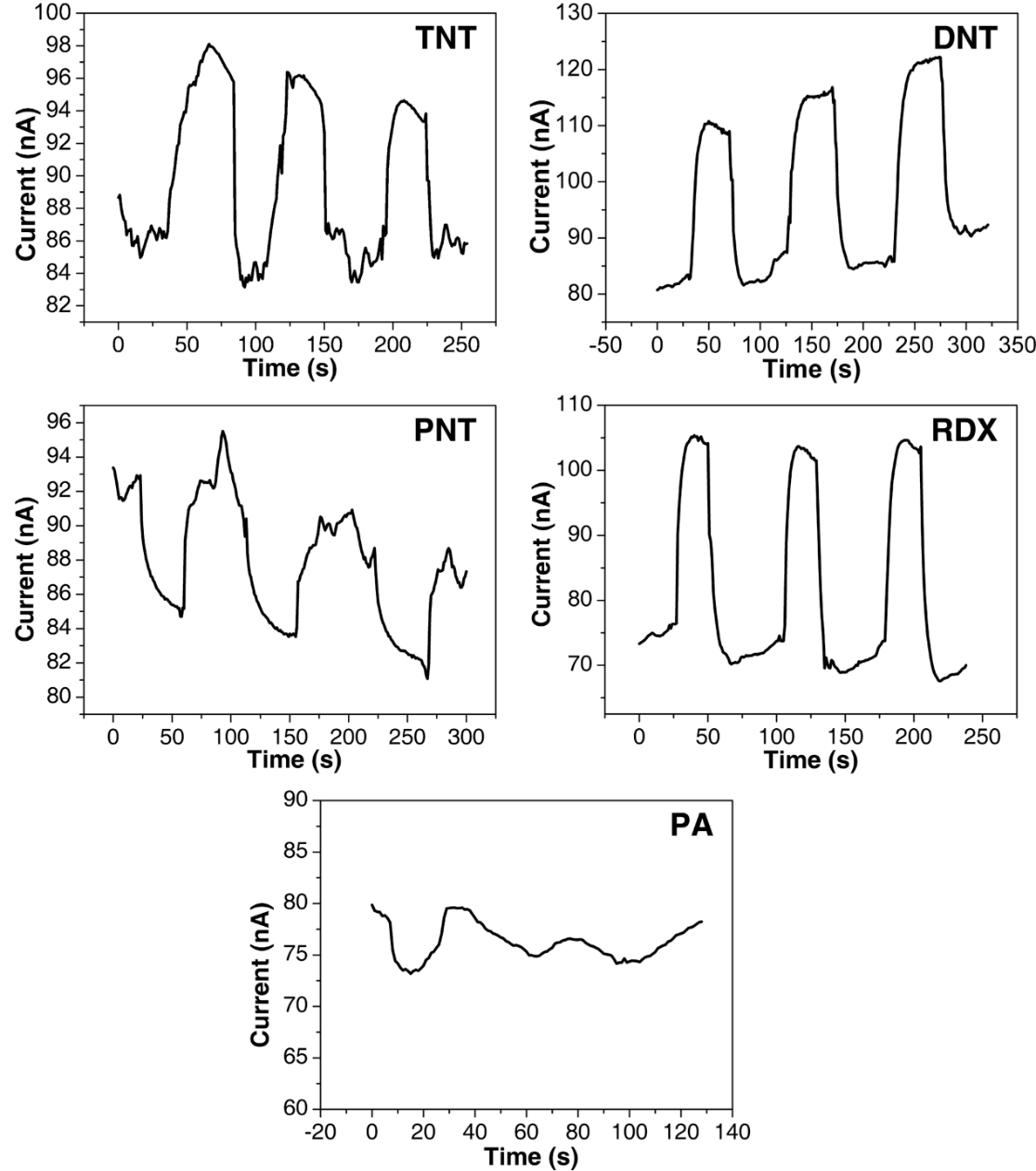


**Supplementary Figure 1.** The current curves of sensor 1 (0 HF) in the gas sensory array towards 6 military explosive vapors (TNT, DNT, PNT, RDX, NT and PA)


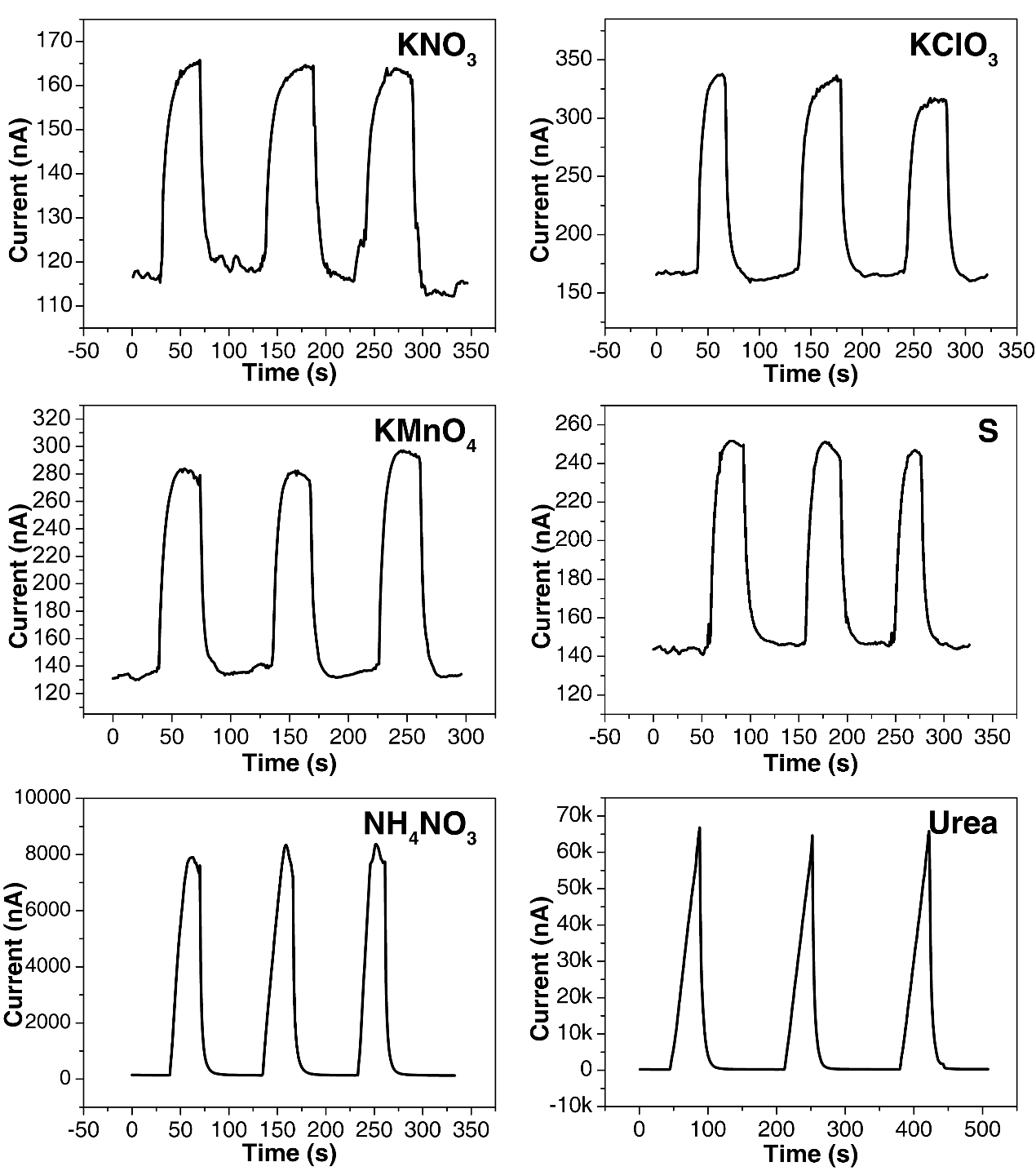


**Supplementary Figure 2.** The current curves of sensor 1 (0 HF) in the gas sensory array towards 6 improvised explosive vapors (KNO_3_, KClO_3_, KMnO_4_, S, NH_4_NO_3_ and urea)


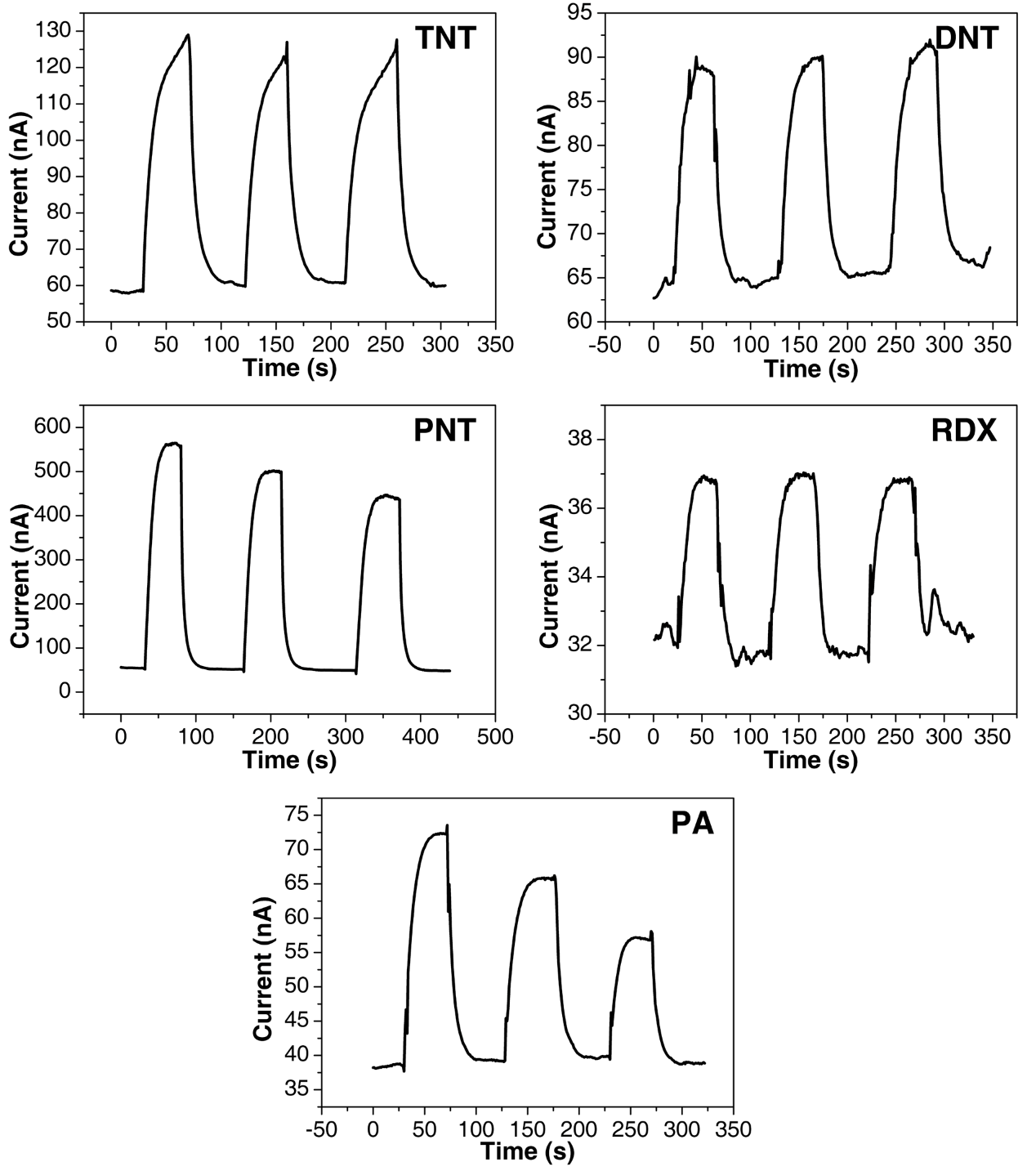


**Supplementary Figure 3.** The current curves of sensor 2 (0.25 HF) in the gas sensory array towards 6 military explosive vapors (TNT, DNT, PNT, RDX, NT and PA)


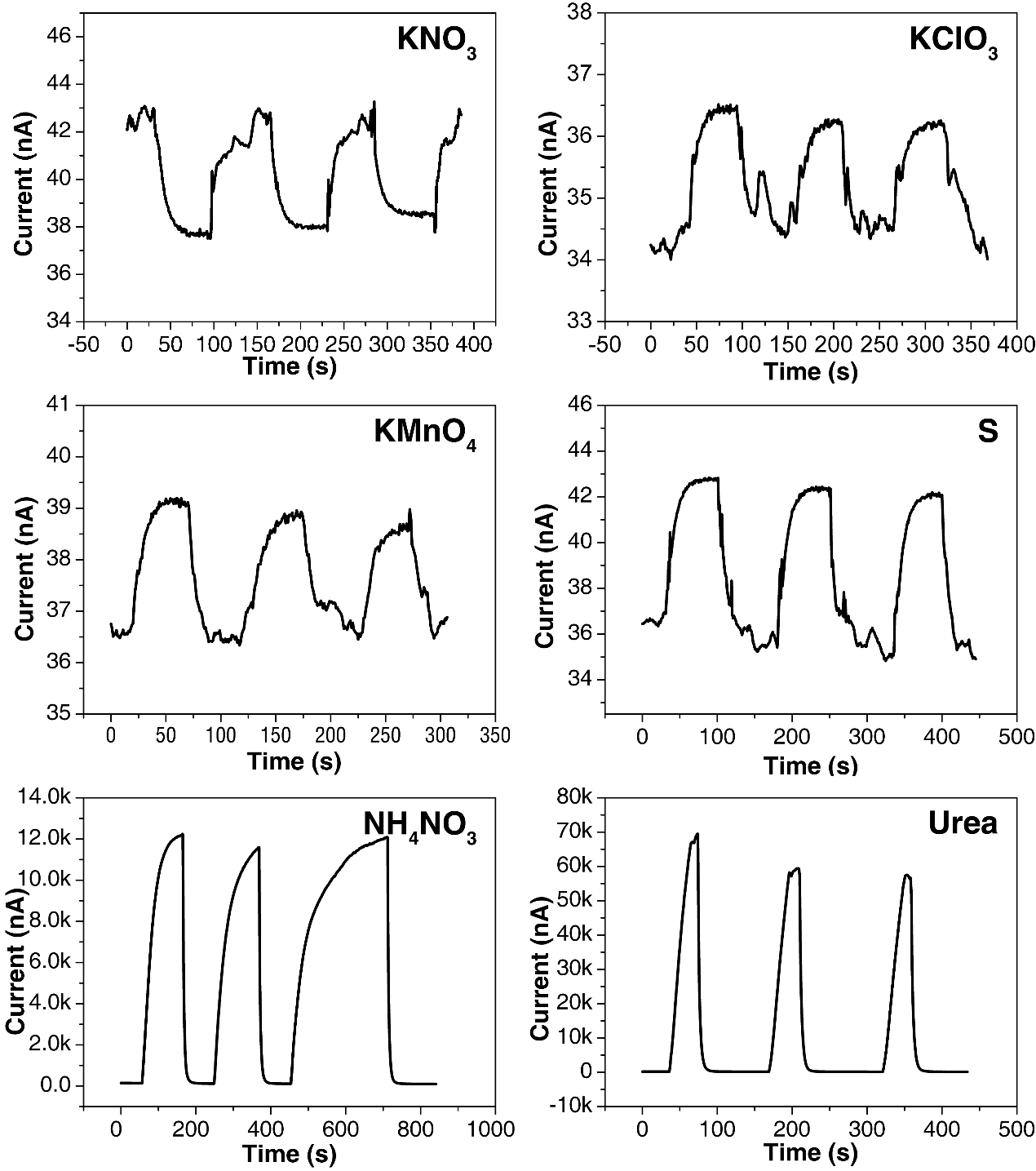


**Supplementary Figure 4.** The current curves of sensor 2 (0.25 HF) in the gas sensory array towards 6 improvised explosive vapors (KNO_3_, KClO_3_, KMnO_4_, S, NH_4_NO_3_ and urea)


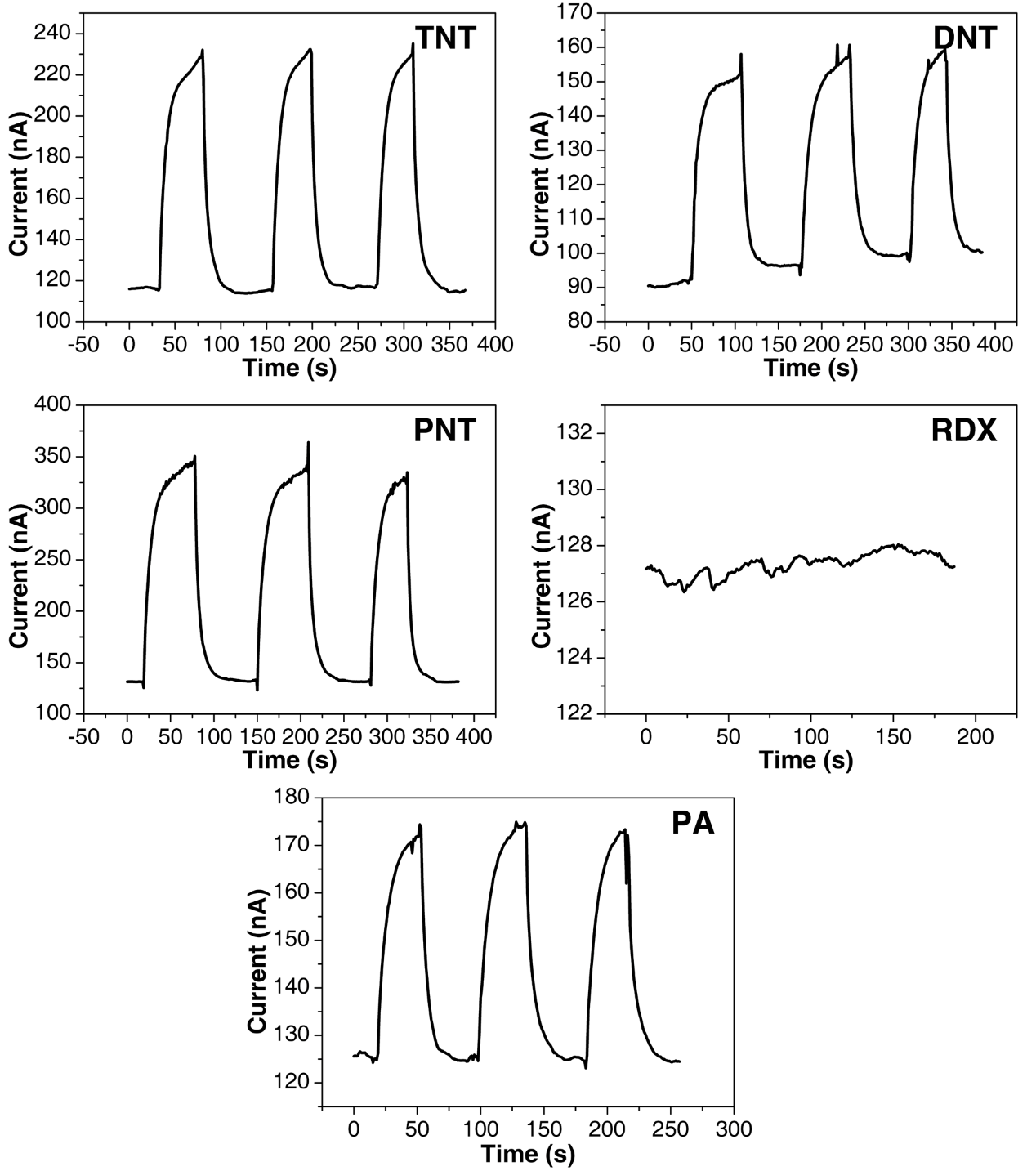


**Supplementary Figure 5.** The current curves of sensor 3 (0.5 HF) in the gas sensory array towards 6 military explosive vapors (TNT, DNT, PNT, RDX, NT and PA)


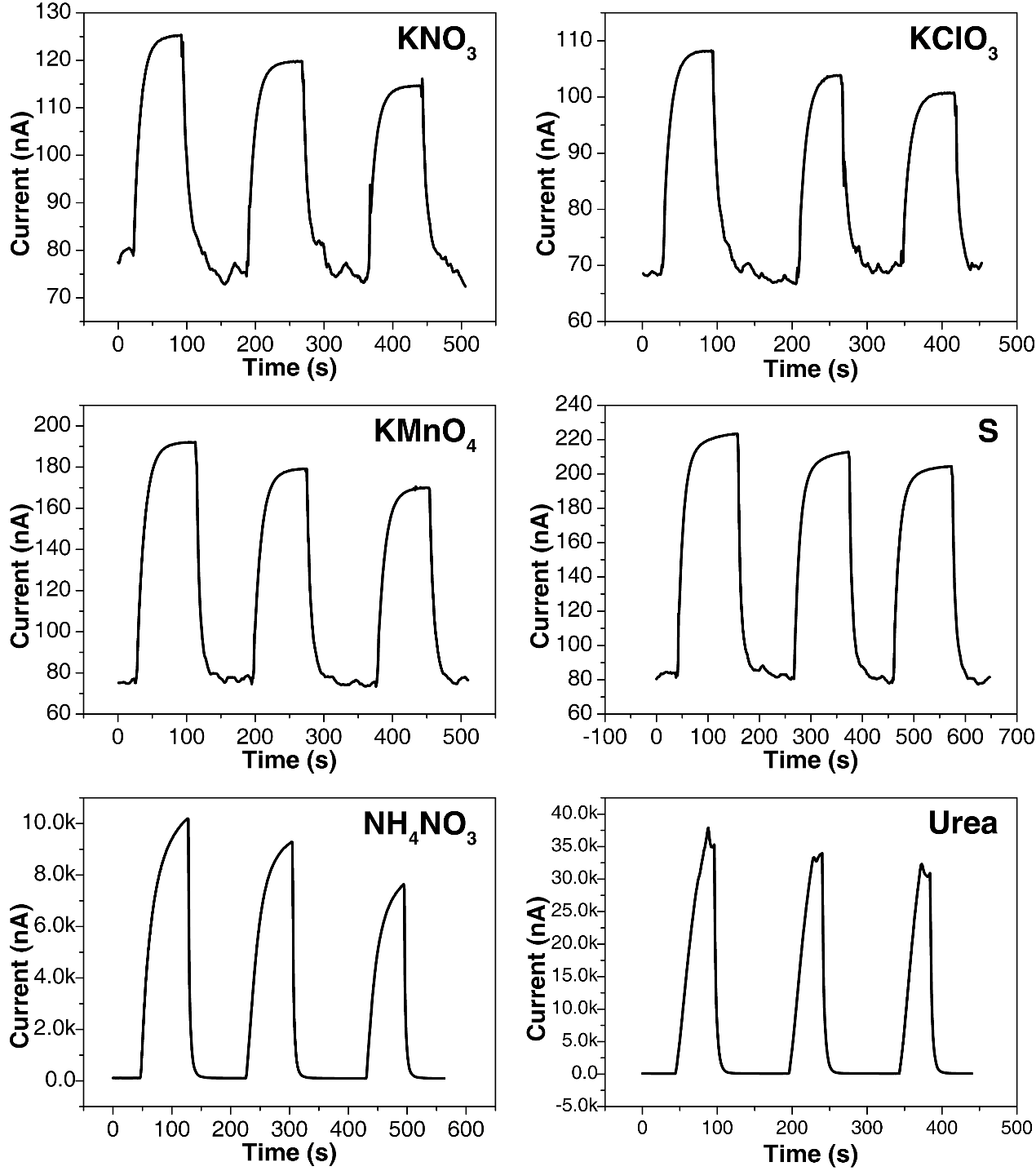


**Supplementary Figure 6.** The current curves of sensor 3 (0.5 HF) in the gas sensory array towards 6 improvised explosive vapors (KNO_3_, KClO_3_, KMnO_4_, S, NH_4_NO_3_ and urea)


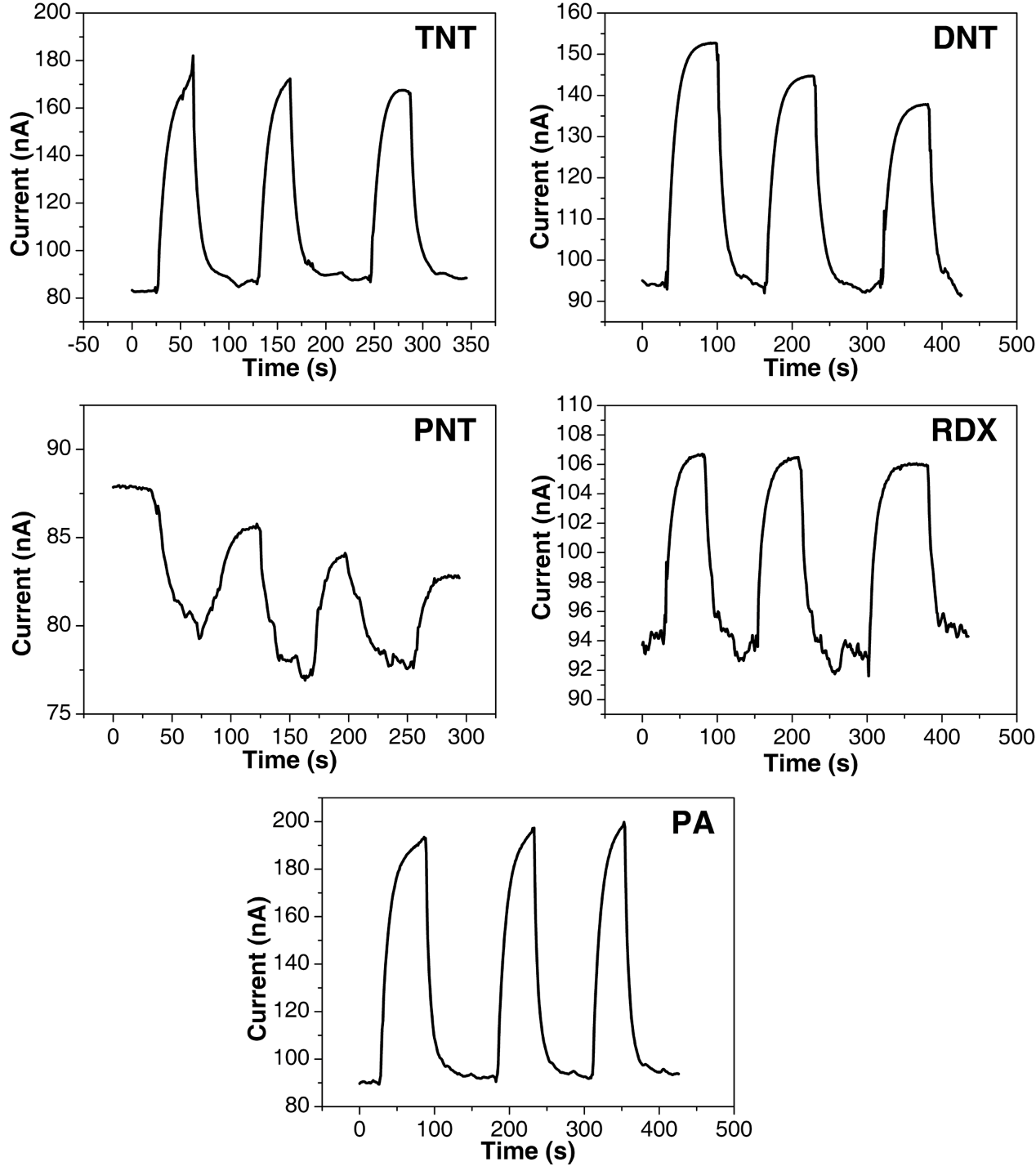


**Supplementary Figure 7.** The current curves of sensor 4 (0.75 HF) in the gas sensory array towards 6 military explosive vapors (TNT, DNT, PNT, RDX, NT and PA)


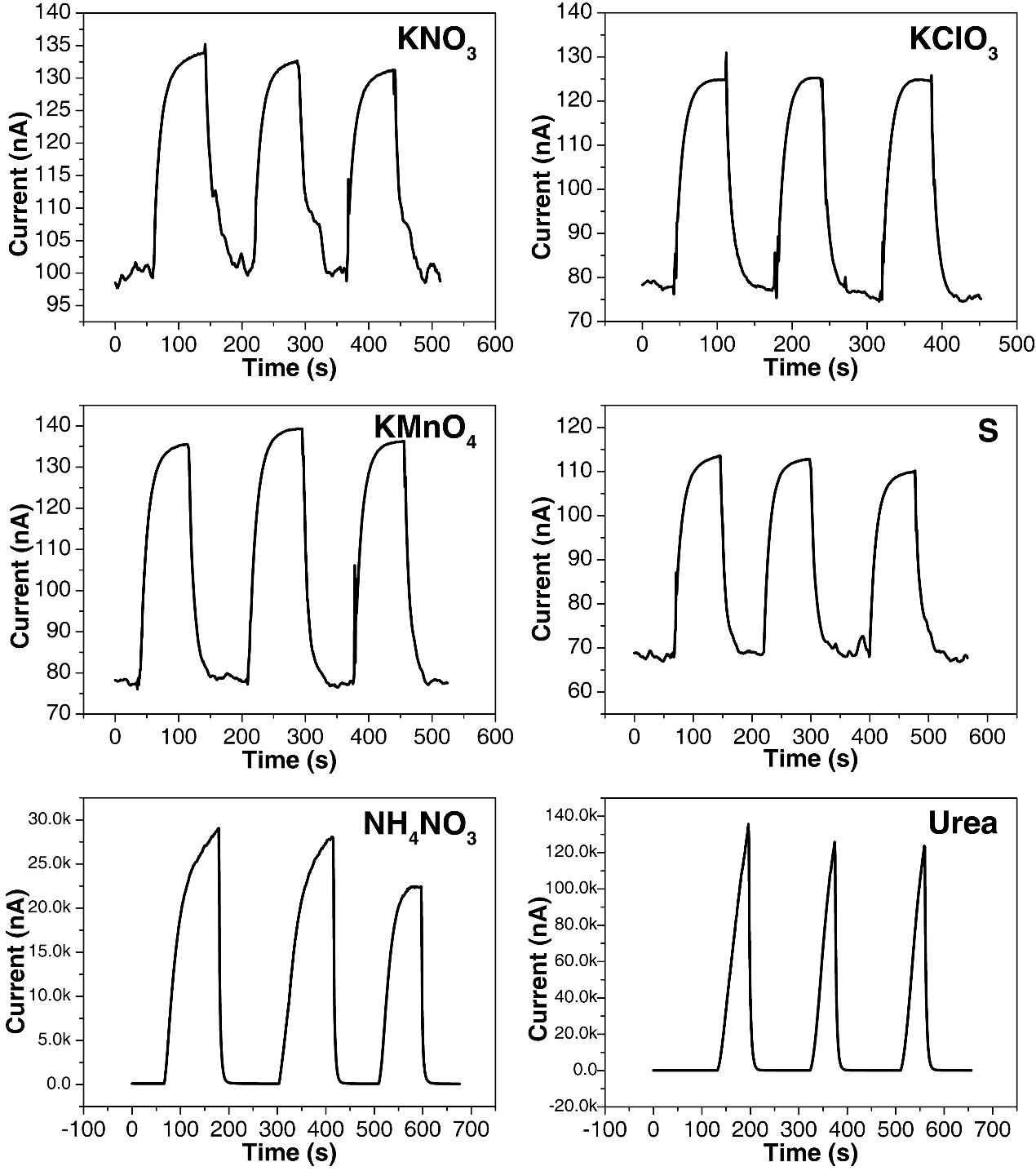


**Supplementary Figure 8.** The current curves of sensor 4 (0.75 HF) in the gas sensory array towards 6 improvised explosive vapors (KNO_3_, KClO_3_, KMnO_4_, S, NH_4_NO_3_ and urea)


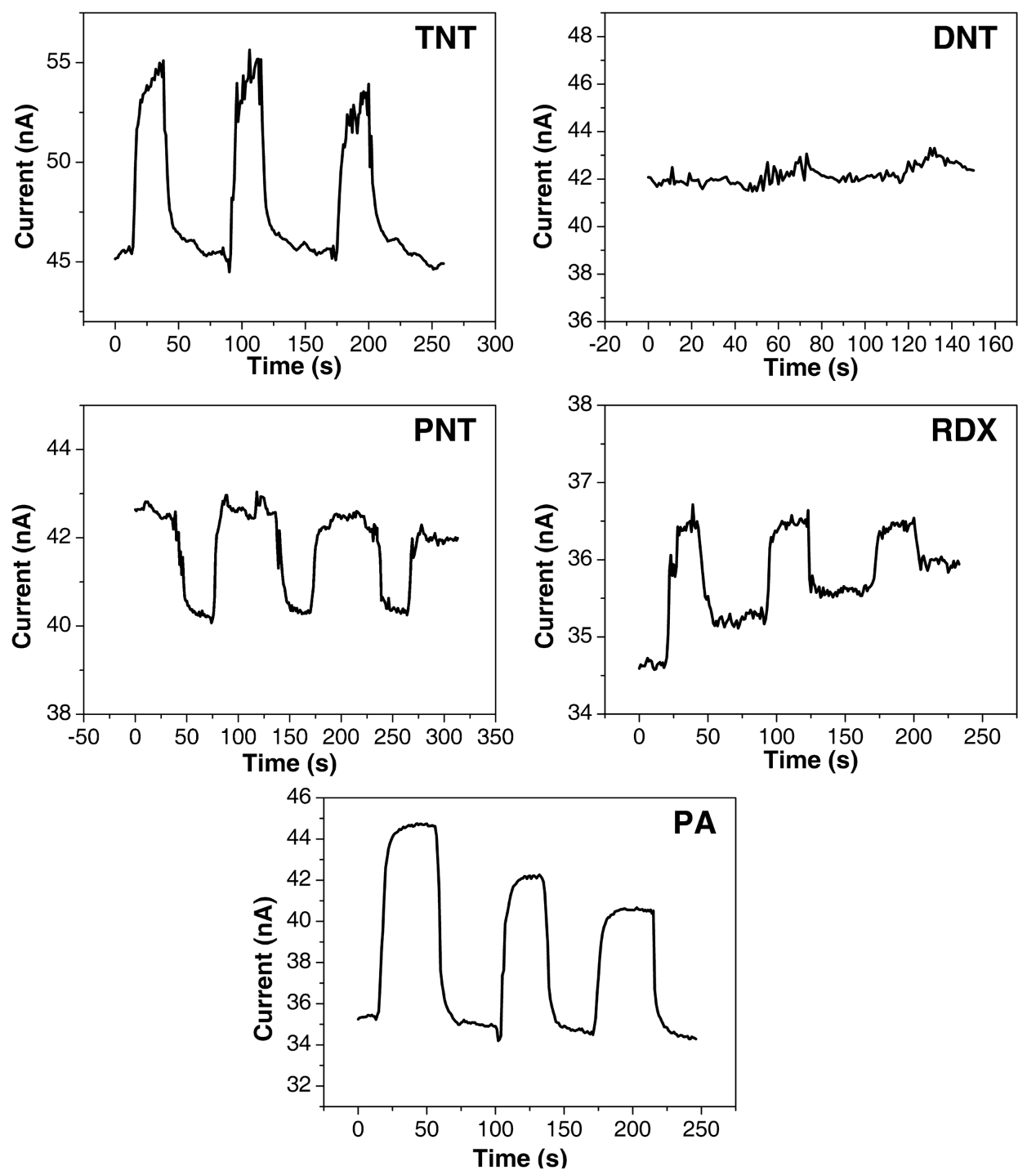


**Supplementary Figure 9.** The current curves of sensor 5 (1 HF) in the gas sensory array towards 6 military explosive vapors (TNT, DNT, PNT, RDX, NT and PA)


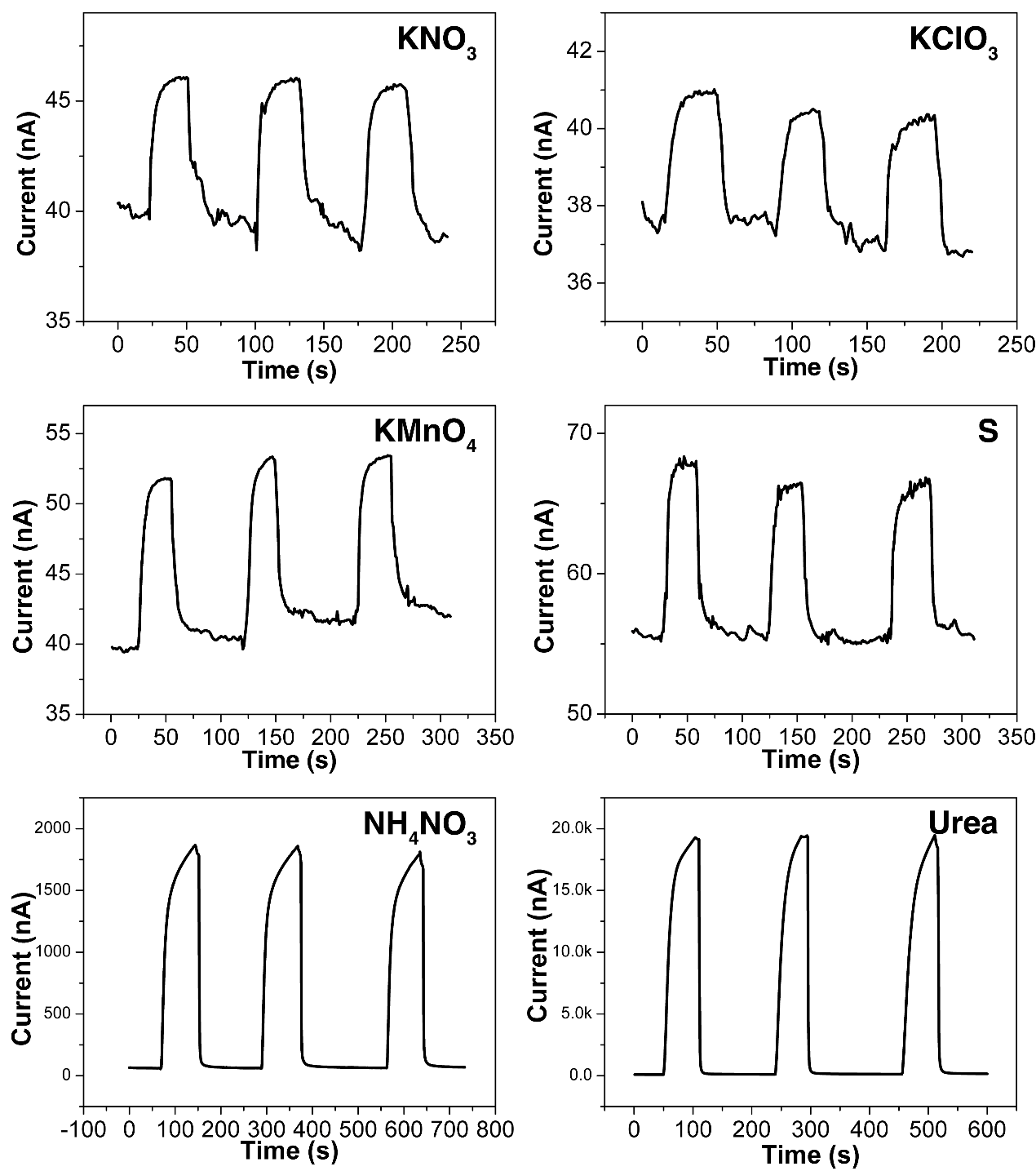


**Supplementary Figure 10.** The current curves of sensor 5 (1 HF) in the gas sensory array towards 6 improvised explosive vapors (KNO_3_, KClO_3_, KMnO_4_, S, NH_4_NO_3_ and urea)


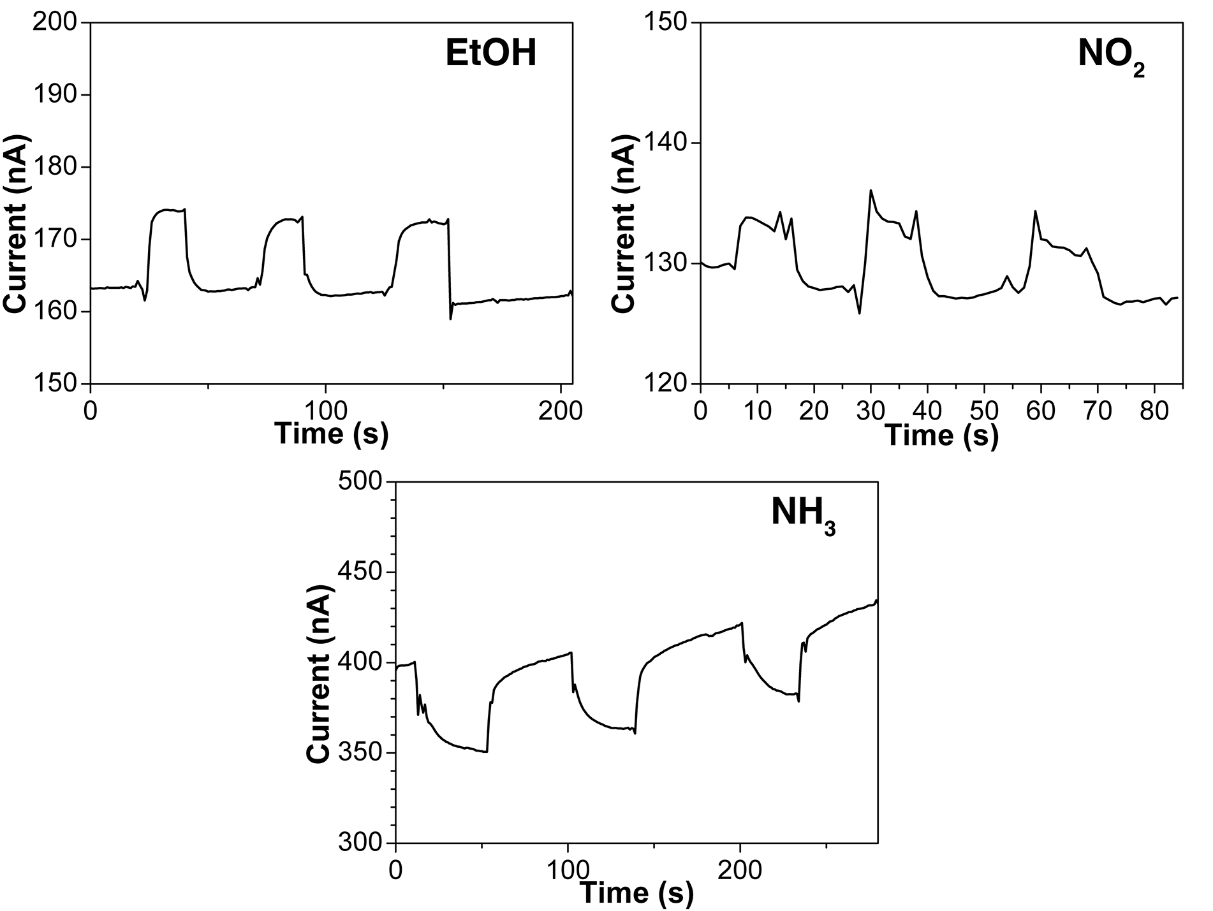


**Supplementary Figure 11.** The current curves of sensor 1 (0 HF) in the gas sensory array towards 3 interfering gases with concentration of 1 ppm (EtOH, NO_2_ and NH_3_)


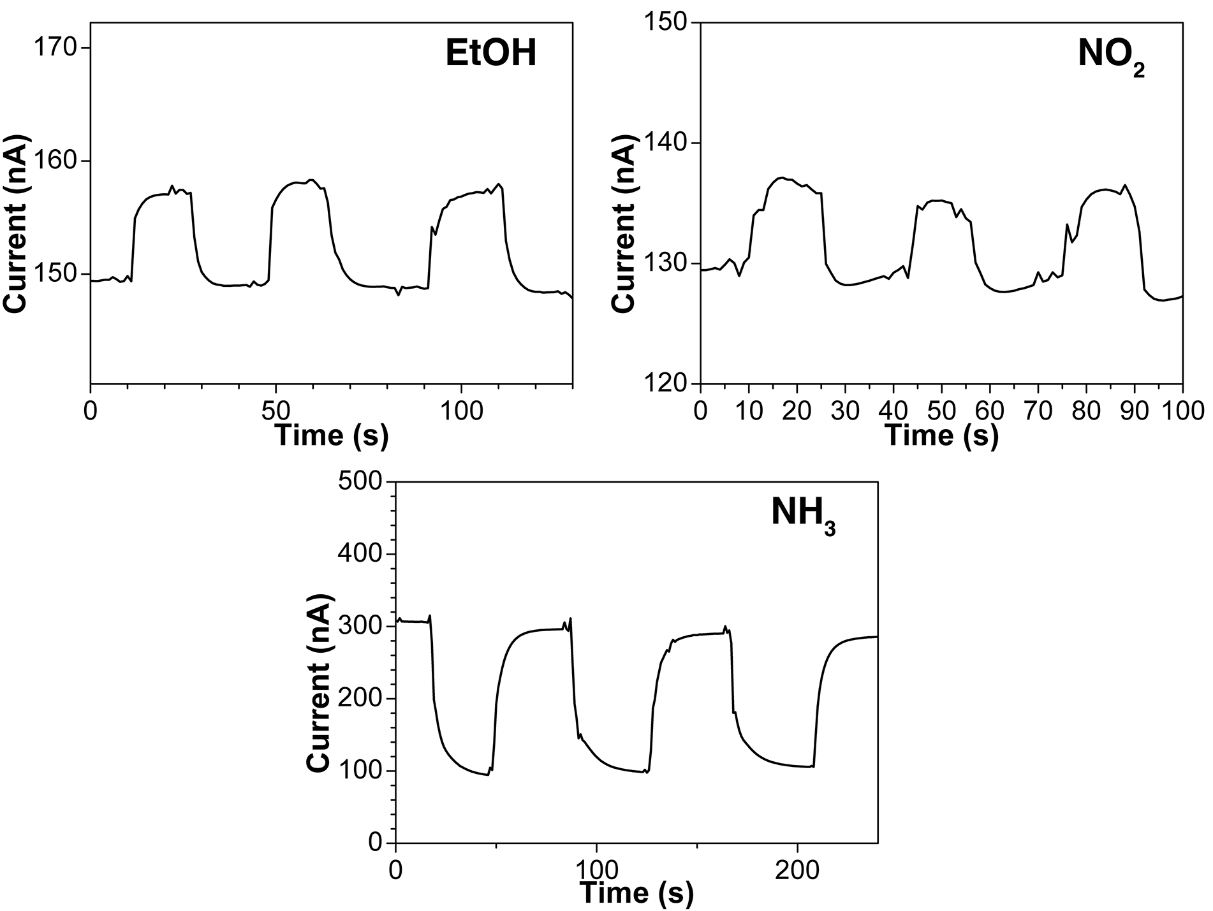


**Supplementary Figure 12.** The current curves of sensor 2 (0.25 HF) in the gas sensory array towards 3 interfering gases with concentration of 1 ppm (EtOH, NO_2_ and NH_3_)


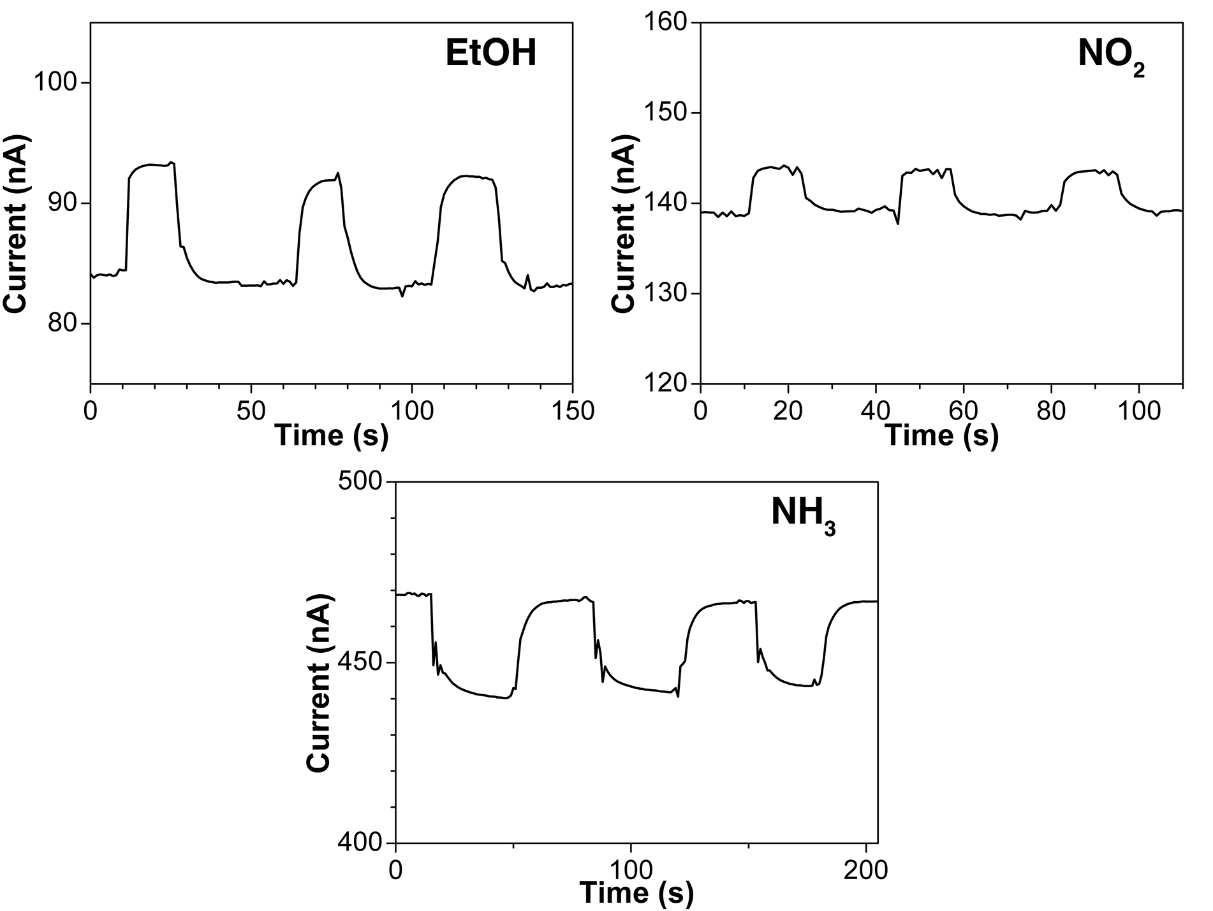


**Supplementary Figure 13.** The current curves of sensor 3 (0.5 HF) in the gas sensory array towards 3 interfering gases with concentration of 1 ppm (EtOH, NO_2_ and NH_3_)


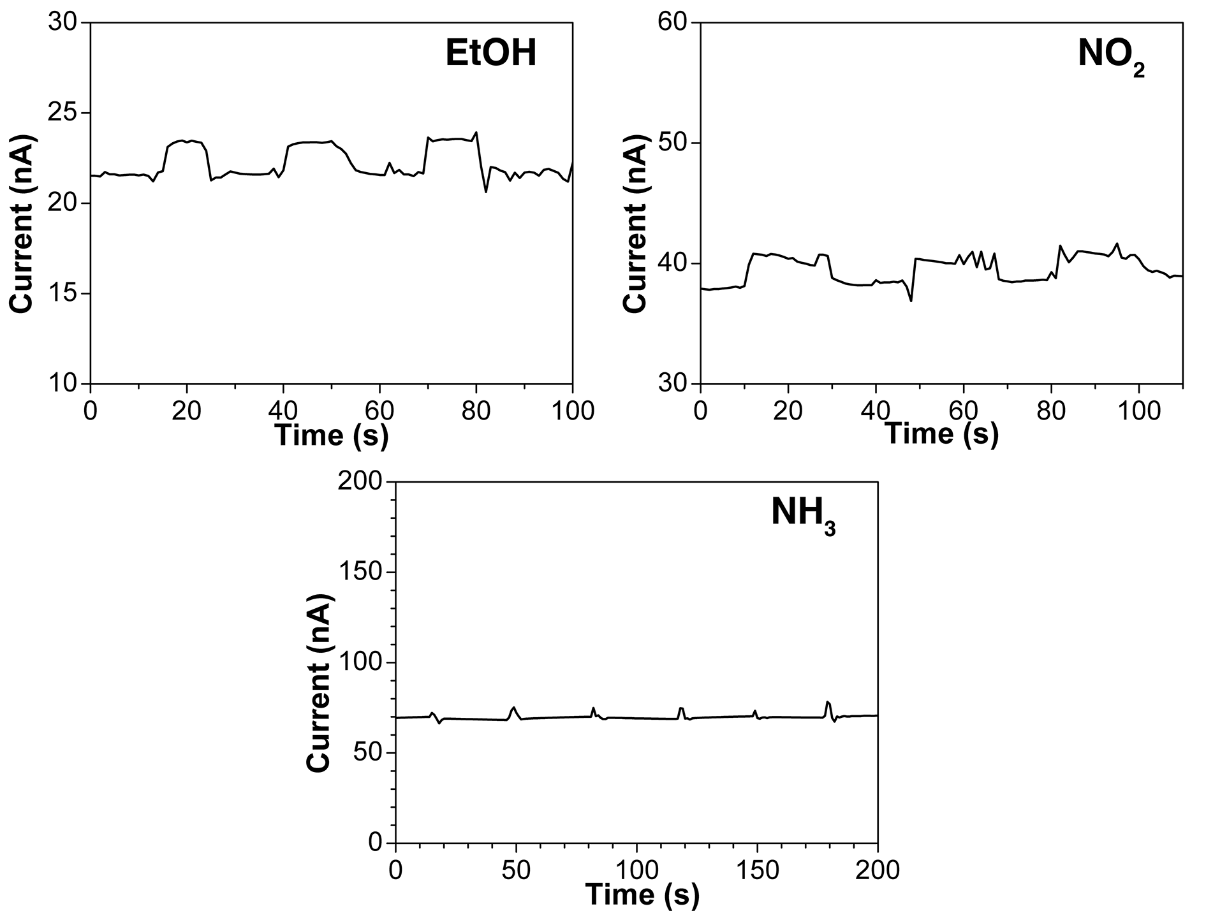


**Supplementary Figure 14.** The current curves of sensor 4 (0.75 HF) in the gas sensory array towards 3 interfering gases with concentration of 1 ppm (EtOH, NO_2_ and NH_3_)


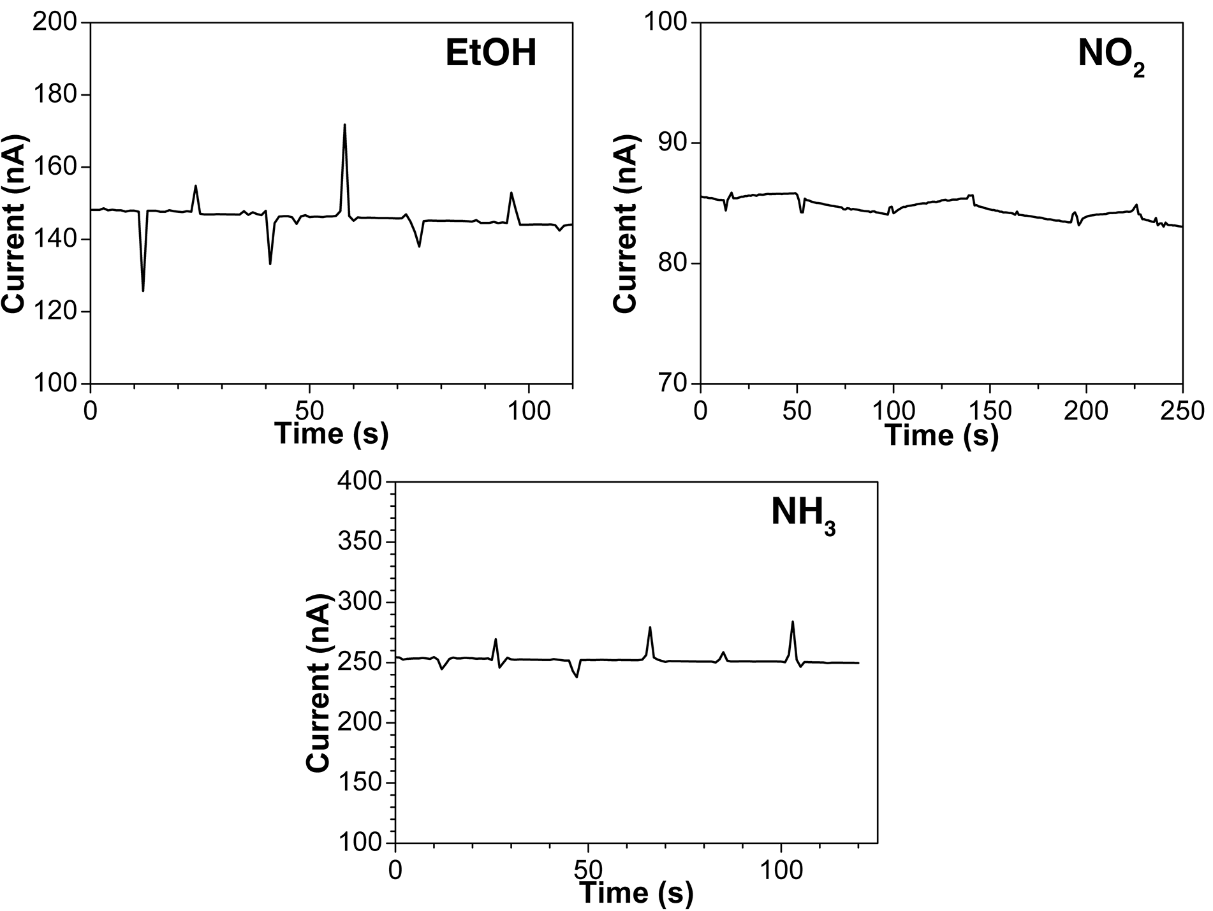


**Supplementary Figure 15.** The current curves of sensor 5 (1 HF) in the gas sensory array towards 3 interfering gases with concentration of 1 ppm (EtOH, NO_2_ and NH_3_)

**Supplementary Table 1** Room-temperature saturated vapor concentrations of 11 analytes

| **Analyte** | **Vaper**  **concentration** | **Reference** | **note** |
| --- | --- | --- | --- |
| TNT | 9 ppb | (Ewing et al., 2013) |  |
| DNT | 411 ppb |  |  |
| PNT | 647 ppb |  |  |
| PA | 0.97 ppb |  |  |
| RDX | 4.9 ppt |  |  |
| NH_4_NO_3_ | 14.7 ppb |  |  |
| S | 2 ppb | (Lyons, 2011) |  |
| urea | 9 ppt | (Krasulin et al., 1987) | Note1 |
| KNO_3_ | **--** |  | Note2 |
| KClO_3_ | **--** |  |  |
| KMnO_4_ | **--** |  |  |

Note1: In the case of urea, the room temperature saturated vapor pressure of about 9 ppt could be obtained by extrapolating the curve of its saturated vapor pressure as a function of temperature within the range of 56-130 oC: $\ln\left( p \right)=-\left( 11755\pm268 \right)T^{-1}+(32.472\pm0.716)$ to the room temperature (298.15 K).(Krasulin et al., 1987) Besides, due to its high hygroscopicity, the humidity surrounding urea would be affected largely and a thin layer of aqueous solution could be formed on the surface of urea particles. The urea in the solution can decompose to NH_3_ and CO_2_ at room temperature. (Shaw and Bordeaux, 1955; Schaber et al., 2004) Therefore, the gas sensing signal of urea is a combination of three aspects: 1) the urea vapor; 2) surrounding humidity; and 3) the decomposition products.

Note2: For KNO_3_, KClO_3_, and KMnO_4_, owing to their ionic crystal nature, there is no vapor pressure data available at room temperature. Moreover, they all undergo decomposition and release oxygen at high temperature.

For KNO_3_, the decomposition occurs at around 650 oC.(Stern, 1972)

$$2KNO_{3}\to K_{2}O+2NO_{2}+1/2O_{2}$$

For KClO_3_, the decomposition occurs at around 400 oC. (Bostrup et al., 1962)

$$2KClO_{3}\to2KCl+3O_{2}$$

For KMnO_4_, the decomposition occurs at around 250 ^o^C.(Herbstein et al., 1971)

$$10\mathrm{KMnO}_{4}\to2.65K_{2}\mathrm{Mn}O_{4}+\left( 2.35K_{2}O,7.35MnO_{2.05} \right)+6O_{2}$$

However, according to thermodynamic theory, the decomposition could only occur at room temperature with an extremely slow reaction rate resulting negligible vapor pressure of the gaseous decomposition products. Therefore, neither the vapor of themselves nor their decomposition products is responsible for the gas sensing signal. In fact, we believe that some tiny crystal clusters containing several unit cells float around in the vapor of these explosives, which could interact with the surface of sensing materials and hence are responsible for the electric signal change.

**Supplementary Table 2** Comparison of different vapor electrical sensors and TiO_2_ nanosheets-based gas sensors array

| Sensing materials | Analytes and concentration | Responses | Ref. |
| --- | --- | --- | --- |
| ZnO nanowires  SWNT | TNT 60 ppb,  TNT, 8 ppb | 20%  5% | (Chen et al., 2010) |
| Titania(B) nanowires | TNT, 9ppb  DNT, 180 ppb | 47%  38% | (Wang et al., 2011) |
| GaN Nanowires-Titania Nanocluster Hybrids | TNT, 9ppb  DNT, 180 ppb  PNT, 647 ppb | 5%  20%  20% | (Aluri et al., 2013) |
| Covalently Functionalized  SWNT | Nitromethane, 57 ppm  Cyclohexanone, 57 ppm | 0.1%  0.5% | (Schnorr et al., 2013) |
| Noncovalent metalloporphyin decorated SWNT | Toluene, 1000 ppm | 8.1% | (Liu et al., 2015) |
| n-type organic nanoribbons | DNT, 0.1 ppm  PNT, 100 ppm | 40%  16% | (Che et al., 2010) |
| SiNWs array/TiO_2_  /rGO Schottky  heterojunction | TNT, 9 ppb  DNT, 180 ppb  PNT, 647 ppb  RDX, 4.9 ppt  PA, 0.97 ppb | 6.3%  40%  56%  9%  4% | (Yang et al., 2015) |
| TiO_2_ nanosheets | TNT, 9 ppb  DNT, 180 ppb  PNT, 647 ppb  RDX, 4.9 ppt  PA, 0.97 ppb | 115.6%  65.5%  830.0%  40.0%  115.0% | This work |

**Supplementary Table 3** Comparison of different detection techniques of detecting military explosives

| Detecting techniques and materials | Analytes and concentration | Performance index | Ref. |
| --- | --- | --- | --- |
| Fluorescent sensor, ZnO nanorods | TNT, 9 ppb | Quenching efficiency, 55%, 200 s | (Zhu et al., 2011) |
| Fluorescent sensor | RDX, 0.012 M | Flourescence increase, 80 fold, 10 s | (Andrew and Swager, 2007) |
| Fluorescent sensor, oligo(diphenylsilane)s | TNT, 9 ppb  DNT, 180 ppb | TNT quenching efficiency, 83%, 30 s  DNT quenching efficiency, 94%, 30 s | (He et al., 2009) |
| Fluorescent sensor | DNT, 0.008 M  PNT, 0.008 M | DNT, Stern-Volmer constants, 49 M^-1^, 7 ns  PNT, Stern-Volmer constants, 38 M^-1^, 7 ns | (Olley et al., 2010) |
| SERS, silver nanoparticles | TNT, 1.4 ppm | 2 s | (Wang et al., 2014) |
| Ion mobility spectrometer | TNT, 10 ng | 14 s | (Zhou et al., 2015) |
| Chemiresistive sensors array | TNT, 9 ppb  DNT, 180 ppb  PNT, 647 ppb  RDX, 4.9 ppt  PA, 0.97 ppb | Less than 30 s | This work |

**Supplementary Table 4** Comparison of detection techniques for improvised explosives

| Analytical method | Analytes and concentration | Consuming time | Ref. |
| --- | --- | --- | --- |
| Ion mobility spectrometer (IMS) | KNO_3_，KClO_3_，KClO_4_, 10^-12^ g | 5 s plus sampling time | (Peng et al., 2014) |
| Capillary electrophoresis (CE) | 15 anions, 0.24-1.15 mg/L  12 cations, 0.11-2.30 mg/L | 9 min  9.5 min | (Herbstein et al., 1971) |
| Ion chromatography (IC) | 18 anions, 2-27.4 ppb  12 cations, 13-115 ppb | Unknown  Unknown | (Johns et al., 2008) |
| Chemiresistive sensors array | KNO_3_, KClO_3_, KMnO_4_, S, NH_4_NO_3_, urea (room temperature saturated vapor) | Less than 75 s | This work |

**References**

Aluri, G.S., Motayed, A., Davydov, A.V., Oleshko, V.P., Bertness, K.A., and Rao, M.V. (2013). Nitro-Aromatic Explosive Sensing Using GaN Nanowire-Titania Nanocluster Hybrids. *IEEE Sens. J.* 13(5), 1883.

Andrew, T.L., and Swager, T.M. (2007). A fluorescence turn-on mechanism to detect high explosives RDX and PETN. *J. Am. Chem. Soc.* 129(23), 7254-7255.

Bostrup, O., Demandt, K., and Hansen, K.O. (1962). The thermal decomposition of KClO_3_. *J. Chem. Edu.* 39(11), 573.

Che, Y., Yang, X., Liu, G., Yu, C., Ji, H., Zuo, J., et al. (2010). Ultrathin n-type organic nanoribbons with high photoconductivity and application in optoelectronic vapor sensing of explosives. *J. Am. Chem. Soc.* 132(16), 5743-5750.

Chen, P.C., Sukcharoenchoke, S., Ryu, K., Gomez de Arco, L., Badmaev, A., Wang, C., et al. (2010). 2, 4, 6‐Trinitrotoluene (TNT) Chemical Sensing Based on Aligned Single‐Walled Carbon Nanotubes and ZnO Nanowires. *Adv. Mater.* 22(17), 1900-1904.

Ewing, R.G., Waltman, M.J., Atkinson, D.A., Grate, J.W., and Hotchkiss, P.J. (2013). The vapor pressures of explosives. *TrAC Trends in Analytical Chemistry* 42, 35-48.

He, G., Zhang, G., Lü, F., and Fang, Y. (2009). Fluorescent film sensor for vapor-phase nitroaromatic explosives via monolayer assembly of oligo (diphenylsilane) on glass plate surfaces. *Chem. Mater.* 21(8), 1494-1499.

Herbstein, F.H., Ron, G., and Weissman, A. (1971). The thermal decomposition of potassium permanganate and related substances. Part I. Chemical aspects. *Journal of the Chemical Society A: Inorganic, Physical, Theoretical* (0), 1821-1826.

Johns, C., Shellie, R.A., Potter, O.G., O’Reilly, J.W., Hutchinson, J.P., Guijt, R.M., et al. (2008). Identification of homemade inorganic explosives by ion chromatographic analysis of post-blast residues. *J. Chromatogr. A* 1182(2), 205-214.

Krasulin, A.P., Kozyro, A.A., and Kabo, G.Y. (1987). Saturation Vapor-pressure of Urea in the temperature-range 329-403-K. *J. Appl. Chem. of the Ussr* 60(1), 96-99.

Liu, S.F., Moh, L.C., and Swager, T.M. (2015). Single-Walled Carbon Nanotube–Metalloporphyrin Chemiresistive Gas Sensor Arrays for Volatile Organic Compounds. *Chem. Mater.* 27(10), 3560-3563.

Lyons, J.R. (2011). An estimate of the equilibrium speciation of sulfur vapor over solid sulfur and implications for planetary atmospheres. *J. of Sulfur Chem.* 29(3-4), 269-279.

Olley, D.A., Wren, E.J., Vamvounis, G., Fernee, M.J., Wang, X., Burn, P.L., et al. (2010). Explosive sensing with fluorescent dendrimers: the role of collisional quenching. *Chem. Mater.* 23(3), 789-794.

Peng, L., Hua, L., Wang, W., Zhou, Q., and Li, H. (2014). On-site Rapid Detection of Trace Non-volatile Inorganic Explosives by Stand-alone Ion Mobility Spectrometry via Acid-enhanced Evaporization. *Sci. Rep.* 4, 6631.

Schaber, P.M., Colson, J., Higgins, S., Thielen, D., Anspach, B., and Brauer, J. (2004). Thermal decomposition (pyrolysis) of urea in an open reaction vessel. *Thermochimica Acta* 424(1-2), 131-142.

Schnorr, J.M., van der Zwaag, D., Walish, J.J., Weizmann, Y., and Swager, T.M. (2013). Sensory Arrays of Covalently Functionalized Single‐Walled Carbon Nanotubes for Explosive Detection. *Adv. Funct. Mater.* 23(42), 5285-5291.

Shaw, W.H.R., and Bordeaux, J.J. (1955). The Decomposition of Urea in Aqueous Media. *Journal of the American Chemical Society* 77(18), 4729-4733.

Stern, K.H. (1972). High temperature properties and decomposition of inorganic salts part 3, nitrates and nitrites. *Journal of Physical and Chemical Reference Data* 1, 747-772.

Wang, D., Chen, A., Jang, S.-H., Yip, H.-L., and Jen, A.K.-Y. (2011). Sensitivity of titania (B) nanowires to nitroaromatic and nitroamino explosives at room temperature via surface hydroxyl groups. *J. Mater. Chem.* 21(20), 7269-7273.

Wang, J., Yang, L., Liu, B., Jiang, H., Liu, R., Yang, J., et al. (2014). Inkjet-printed silver nanoparticle paper detects airborne species from crystalline explosives and their ultratrace residues in open environment. *Anal. Chem.* 86(7), 3338-3345.

Yang, Z., Dou, X., Zhang, S., Guo, L., Zu, B., Wu, Z., et al. (2015). A High‐Performance Nitro‐Explosives Schottky Sensor Boosted by Interface Modulation. *Adv. Funct. Mater.*, 4039-4048.

Zhou, Q., Peng, L., Jiang, D., Wang, X., Wang, H., and Li, H. (2015). Detection of Nitro-Based and Peroxide-Based Explosives by Fast Polarity-Switchable Ion Mobility Spectrometer with Ion Focusing in Vicinity of Faraday Detector. S*ci. Rep.* 5, 10659.

Zhu, D., He, Q., Chen, Q., Fu, Y., He, C., Shi, L., et al. (2011). Sensitivity gains in chemosensing by optical and structural modulation of ordered assembly arrays of ZnO nanorods. *ACS nano* 5(6), 4293-4299.
